# Photobiomodulation and Low-Level Laser Therapy as Complementary Strategies in Diabetes Treatment

**DOI:** 10.3390/ijms27042078

**Published:** 2026-02-23

**Authors:** Natalia Kurhaluk, Vladimir Tomin, Renata Kołodziejska, Halina Tkaczenko

**Affiliations:** 1 Institute of Biology, Pomeranian University in Słupsk, Arciszewski St. 22b, 76-200 Słupsk, Poland; 2 Institute of Exact and Technical Sciences, Pomeranian University in Słupsk, Arciszewski St. 22b, 76-200 Słupsk, Poland; vladimir.tomin@upsl.edu.pl; 3 Department of Medical Biology and Biochemistry, Collegium Medicum in Bydgoszcz, Nicolaus Copernicus University in Toruń, M. Karłowicz St. 24, 85-092 Bydgoszcz, Poland; renatak@cm.umk.pl

**Keywords:** photobiomodulation therapy (PBMT), low-level laser therapy (LLLT), diabetes mellitus, wound healing, neuropathic pain, glycaemic control, oxidative stress, vascular function

## Abstract

Diabetes mellitus is a multifactorial metabolic disorder associated with a number of chronic complications, including neuropathy, impaired wound healing, vascular dysfunction, and metabolic dysregulation. Despite advances in pharmacological treatments and lifestyle interventions, current therapies often fail to prevent or reverse these complications entirely. This narrative review examines the therapeutic potential of laser-based modalities, particularly low-level laser therapy (LLLT) and photobiomodulation therapy (PBMT), as complementary strategies in diabetes management. Analysis of experimental and clinical studies shows that laser therapy can enhance wound healing, alleviate neuropathic pain, improve glycaemic control and insulin sensitivity, modulate inflammatory and oxidative stress pathways, and support vascular function. These effects are primarily mediated through mitochondrial activation, nitric oxide release, angiogenesis, modulation of redox-sensitive transcription factors, and preservation of pancreatic β-cell function. Furthermore, laser therapy exhibits a favourable safety profile with minimal side effects. The review highlights the current challenges, such as the lack of standardised treatment parameters (e.g., wavelength, dosage, and duration) and the limited number of large-scale clinical trials. It emphasises the need for personalised protocols and integration of laser therapy with pharmacological and physiotherapeutic interventions. Continued research and interdisciplinary collaboration are needed to realise the potential of laser therapy as an integral component of comprehensive, evidence-based diabetes care.

## 1. Introduction

In 1960, the first source of coherent monochromatic light in the visible spectrum—a laser—was constructed. It produced light with unique properties that paved the way for future applications in many fields [1]. A few years later, in 1967, Endre Mester in Hungary performed the first demonstration of ‘laser biostimulation’ on mice, opening the door to important applications in medicine, veterinary science, and biology. Since then, the use of coherent (laser) or non-coherent (light-emitting diode, LED) light sources in medical treatment has developed significantly. Currently, low-level laser (or light) therapy (LLLT), often referred to as ‘biostimulation’ or ‘photobiomodulation’ (PBM), is being widely explored in physiotherapy and regenerative medicine in many countries [2]. The mechanism of LLLT involves cellular chromophores absorbing photons, which modulates cellular metabolism and signalling pathways without causing thermal damage. This makes LLLT suitable for delicate tissues and chronic conditions [3,4].

Light therapy is one of the oldest therapeutic methods used by humans. Historically, it was used as heliotherapy in ancient Egypt and later as ultraviolet (UV) therapy. Niels Finsen was awarded the 1903 Nobel Prize in Physiology or Medicine for his work on the therapeutic use of concentrated light in the treatment of lupus vulgaris, which highlights the importance of light therapy [5]. This historical milestone laid the foundation for modern phototherapy, which now encompasses a wide range of light-based modalities, including LLLT. This pioneering work established the basis for modern phototherapy, a field that now encompasses a wide range of techniques, including LLLT, ultraviolet (UV) therapy, and light-emitting diode (LED)-based treatments.

Using lasers and LEDs as light sources is a significant development in modern light-based therapies, particularly LLLT. Unlike other light-based medical treatments, LLLT does not rely on thermal effects or cause tissue ablation. It also differs from photodynamic therapy (PDT), which involves activating exogenous chromophores to generate reactive oxygen species (ROS) [6]. The term ‘low-level’ indicates that LLLT uses low-energy doses sufficient to trigger biological responses but too low to cause thermal damage. This makes LLLT a versatile tool for modulating cellular activity without disrupting tissue integrity. This unique property allows LLLT to modulate cellular activity safely, making it a versatile tool in both experimental and clinical settings [3,4].

LLLT has been applied in four major areas of medicine, veterinary science, and biotechnology: (i) wound healing, tissue repair, and prevention of tissue necrosis; (ii) management of chronic diseases, including inflammation reduction, injury recovery, pain relief, and oedema reduction; (iii) relief of neurogenic pain and the treatment of sensory neurological disorders; (iv) fracture healing and bone regeneration [7]. The broad spectrum of applications is underpinned by the ability of LLLT to influence multiple cellular pathways, including mitochondrial activation, cytokine modulation, angiogenesis, and extracellular matrix remodelling [4].

Diabetes mellitus (DM) has received particular attention in recent research due to its increasing prevalence worldwide. Diabetes and metabolic diseases are among the most pressing global health challenges [8]. Often referred to as ‘diseases of civilisation’, these conditions are associated with unhealthy lifestyles, obesity, physical inactivity, and poor diet [9]. DM is a complex metabolic disorder characterised by chronic hyperglycaemia resulting from defects in insulin secretion, insulin action, or both [10]. Despite advances in pharmacological treatments and lifestyle interventions, management of diabetes remains challenging due to serious complications, such as neuropathy, retinopathy, and impaired wound healing [11,12]. These persistent complications highlight the urgent need for additional therapies that address the underlying pathophysiological mechanisms rather than just controlling blood glucose levels.

Due to the limitations of traditional diabetes treatments, the therapeutic potential of emerging therapies such as laser therapy has been the subject of increasing investigation [13,14]. Studies indicate that LLLT can positively impact the pathophysiology of diabetes by promoting wound healing, modulating inflammation, improving glycemic control, and preserving pancreatic function [15,16,17,18]. These effects are mediated through mitochondrial stimulation, increased nitric oxide release, and modulation of redox-sensitive transcription factors, such as NF-κB and HIF-1α. These transcription factors play crucial roles in cellular metabolism and inflammatory regulation.

Studies have demonstrated that LLLT improves cardiac function and myocardial contractility, lowers blood pressure, and enhances lipid metabolism, oxidative stress regulation, antioxidant defences, haemocoagulation, and microcirculation [19]. In the management of diabetes, LLLT has been investigated for its potential to reduce inflammation, accelerate wound healing, and alleviate symptoms of diabetic neuropathy [20,21]. A notable study conducted by Chatterjee et al. (2019) examined the effectiveness of deep tissue laser therapy (DTLT) in alleviating pain and inflammation and enhancing the quality of life of patients with diabetic peripheral neuropathy [22]. The results showed that DTLT significantly reduced serum levels of monocyte chemoattractant protein-1 (MCP-1) and interleukin-6 (IL-6), which are both key inflammatory markers. The therapy also affected RANTES (regulated on activation, normal T-cell expressed and secreted), although this effect was not statistically significant [22]. These findings support the idea that laser therapy can influence systemic inflammatory responses, which are a key factor in the development of diabetic complications.

This paper explores the impact of laser therapy on diabetes management, focusing particularly on its potential clinical applications. Through a comprehensive review of the current evidence on the effects of laser therapy on wound healing, inflammation regulation, insulin sensitivity, and pancreatic function, we determined its potential as a complementary diabetes treatment. Due to the multifactorial nature of diabetes and its complications, combining photobiomodulation with conventional pharmacological and lifestyle interventions could offer additional or enhanced benefits.

To this end, we conducted a narrative review using several scientific databases, including PubMed, Google Scholar, ScienceDirect, Wiley Online Library, Elsevier’s ScienceDirect, MEDLINE (via PubMed), Embase, and Scopus. This comprehensive literature review was designed to capture a wide range of peer-reviewed studies, including clinical trials, in vivo and in vitro investigations, and mechanistic studies, by including diverse databases. This approach ensured broad coverage of relevant research, enhancing the robustness and reliability of the review.

## 2. Materials and Methods

This narrative review aimed to evaluate the impact of laser therapy on diabetes management, examining its effects on wound healing, inflammation regulation, insulin sensitivity, and pancreatic function. A comprehensive literature search was performed using the following electronic databases: PubMed, Google Scholar, ScienceDirect, Wiley Online Library, Elsevier’s ScienceDirect, MEDLINE (via PubMed), EMBASE, and SCOPUS. The search strategy incorporated a combination of Medical Subject Headings (MeSH) terms and free-text keywords, such as ‘low-level laser therapy’, ‘photobiomodulation’, ‘diabetes’, ‘wound healing’, ‘inflammation’, ‘insulin sensitivity’, and ‘oxidative stress’. Boolean operators (AND, OR) were used to refine the search results and ensure the inclusion of relevant studies across multiple disciplines. Search filters were applied to limit the results to human and animal studies.

The inclusion criteria were (1) peer-reviewed studies published in English from 1960 to 2025; (2) experimental and clinical studies investigating the effects of laser therapy on diabetes management; (3) studies analysing the molecular mechanisms of photobiomodulation in diabetes-related complications; (4) articles reporting quantitative outcomes related to wound healing, inflammatory markers, glycaemic control, or pancreatic function. The exclusion criteria included (1) studies without quantitative data or control groups; (2) research focusing solely on photodynamic therapy or other light-based therapies without LLLT involvement; (3) conference abstracts, editorials, and non-peer-reviewed sources. Studies lacking methodological details or statistical analysis were excluded to maintain the integrity of the synthesis. A qualitative synthesis was conducted to summarise the effects of laser therapy on parameters related to diabetes. Where applicable, two reviewers extracted the data independently and resolved any discrepancies through consensus or consultation with a third reviewer. This approach ensured a balanced and critical evaluation of the available evidence, facilitating the identification of therapeutic trends and highlighting areas in need of further research.

## 3. Characteristics of Different Types of Diabetes

Type 1 diabetes mellitus (DM1T) is a chronic autoimmune disease in which the immune system attacks and destroys beta cells in the pancreas. This results in a complete absence of insulin and the requirement for lifelong insulin therapy [23,24]. DM1T typically manifests in childhood or young adulthood, frequently before the age of 30, accounting for 10–15% of all diabetes cases. If left untreated, it can lead to diabetic ketoacidosis (DKA) due to its rapid onset. DM1T is strongly associated with genetic predisposition, particularly with human leukocyte antigen (HLA) genes located on chromosome 6. These genes play a role in the immune response and susceptibility to viral infections that can trigger autoimmunity. While the exact cause remains unknown, environmental factors, such as viral infections, toxins, and dietary triggers, have been implicated [25]. Recent evidence also suggests that changes in the composition of the gut microbiota may influence the activation of the autoimmune response and the destruction of beta cells, offering potential new avenues for preventive and therapeutic strategies [26]. Globally, the incidence of DM1T is increasing by approximately 3–4% each year, particularly among children under the age of 15, posing an escalating public health challenge [27]. Patients require lifelong monitoring to prevent acute and chronic complications.

The clinical manifestations of DM1T include polyuria (frequent urination), polydipsia (excessive thirst), polyphagia (excessive hunger), and sudden weight loss of up to 10 kg over a short period as well as weakness and fatigue. Over 90% of DM1T patients develop autoantibodies against pancreatic islet cells. DM1T is classified as either autoimmune, which accounts for about 90% of cases and is confirmed by the presence of autoantibodies against glutamic acid decarboxylase (GAD), insulin (IAA), and islet cells (ICA), or idiopathic, which accounts for about 10% of cases and is characterised by the absence of detectable autoantibodies yet still requires insulin therapy [23].

DM2T is the most common form of diabetes, accounting for 90–95% of cases worldwide. It is characterised by insulin resistance and relative insulin deficiency, leading to progressive metabolic dysfunction [28]. Unlike DM1T, DM2T typically develops in adults over the age of 40. However, an increasing number of children and adolescents are being diagnosed due to the rising obesity rates, poor dietary habits, and sedentary lifestyles [29]. It is crucial to detect prediabetes early and implement lifestyle interventions to prevent disease progression in younger populations.

DM2T has a strong genetic component, with several genetic loci identified as increasing the risk of developing the disease [30]. Individuals with DM2T exhibit impaired early phase insulin secretion, resulting in delayed responses to glucose intake and excessive postprandial hyperglycaemia [31]. The key pathophysiological mechanisms include (i) impaired pancreatic β-cell function and inadequate insulin secretion; (ii) increased hepatic glucose production due to insulin resistance; (iii) dyslipidaemia and fatty liver disease due to the dysregulation of lipid metabolism; (iv) chronic low-grade inflammation and oxidative stress further exacerbating insulin resistance [32]. DM2T is frequently associated with obesity, hypertension, and dyslipidaemia, forming metabolic syndrome, which markedly increases the risk of cardiovascular complications [33]. The management of DM2T primarily involves lifestyle modifications, oral hypoglycaemic agents and, in advanced stages, insulin therapy. Emerging pharmacological options, such as glucagon-like peptide-1 (GLP-1) receptor agonists and sodium–glucose cotransporter 2 (SGLT2) inhibitors, have been shown to provide significant cardiovascular and metabolic benefits [34].

It is essential to differentiate between DM1T and DM2T in order to manage them appropriately. As well as assessing fasting and postprandial glycaemia, specific biomarkers are used: (i) C-peptide levels reflect endogenous insulin production. They are low or undetectable in DM1T but normal to high in DM2T. (ii) Autoantibody tests (e.g., GAD, ICA, and IAA) can confirm autoimmune DM1T. (iii) An oral glucose tolerance test (OGTT) can assess glucose metabolism and insulin response [35]. A key indicator of long-term glycaemic control in both types of diabetes is glycated haemoglobin (HbA1c), which provides an estimate of average blood glucose levels over the previous two to three months. Maintaining HbA1c levels below 7.0% is a key diabetes management goal [36]. The use of continuous glucose monitoring (CGM) systems alongside HbA1c is becoming more commonplace, as they provide real-time insights into glycaemic variability and hypoglycaemic episodes [35].

Gestational diabetes mellitus (GDM) affects 5–10% of pregnancies, typically being diagnosed between weeks 24 and 28 of gestation. It is caused by hormonal changes that lead to maternal insulin resistance, a condition that is often exacerbated by pre-existing risk factors, such as obesity and a family history of diabetes [37]. While GDM usually resolves after childbirth, women affected by this DM type are seven times more likely to develop type 2 diabetes later in life [38]. The complications of GDM include (i) macrosomia (large birth weight), which increases the risk of birth trauma; (ii) neonatal hypoglycaemia due to excessive foetal insulin production; (iii) an increased risk of pre-eclampsia and caesarean section. Universal screening for GDM and strict glycaemic control during pregnancy can significantly reduce maternal and neonatal complications. Postpartum follow-up involving lifestyle counselling is also essential in order to mitigate the risk of developing long-term T2DM [39].

Other forms of diabetes include (i) maturity-onset diabetes of the young (MODY), a monogenic form of diabetes resulting from genetic mutations affecting β-cell function [40]; (ii) latent autoimmune diabetes in adults (LADA), which is a slowly progressive autoimmune disease displaying characteristics of both type 1 and type 2 diabetes; (iii) secondary diabetes, which is caused by pancreatic disease (e.g., chronic pancreatitis or cystic fibrosis), certain medications (e.g., corticosteroids or antipsychotics), or endocrinopathies (e.g., Cushing’s syndrome or acromegaly) [41]. Accurate classification is crucial because misdiagnosis can result in a wrong treatment being given and an increased risk of complications.

Proper differentiation and early intervention can reduce the risk of long-term complications, such as nephropathy, retinopathy, neuropathy, and cardiovascular disease, thereby improving patients’ life expectancy and quality of life [24,32]. A multidisciplinary team involving endocrinologists, dietitians, diabetes educators, and other healthcare professionals is essential to ensure comprehensive, personalised care for all types of diabetes.

## 4. Molecular Mechanisms and Therapeutic Perspectives in Diabetes and Metabolic Diseases

**Figure 1:**
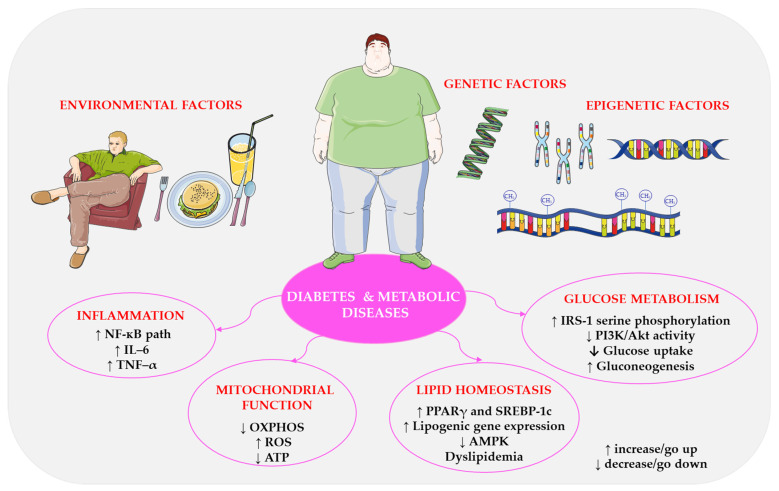
Molecular mechanisms involved in the pathogenesis of diabetes and metabolic diseases. The figure illustrates the complex interplay between genetic, epigenetic, and environmental factors driving metabolic dysfunction. Impaired insulin signaling (IRS-PI3K/Akt, GLUT4), activation of NF-κB, and increased pro-inflammatory cytokines (TNF-α, IL-6) promote insulin resistance and oxidative stress. Dysregulation of PPARγ, SREBP-1c, and AMPK contributes to lipid imbalance, hepatic steatosis, and reduced β-oxidation. Mitochondrial dysfunction—characterized by excessive ROS production, mtDNA damage, and decreased biogenesis—leads to energy deficits and β-cell apoptosis. These processes are further modulated by epigenetic mechanisms, including DNA methylation, histone modifications, and microRNAs, influenced by diet, gut microbiota, and stress, ultimately creating a vicious cycle of metabolic dysregulation. Abbreviations: Akt—protein kinase B (PKB); AMPK—AMP-activated protein kinase; ATP—adenosine triphosphate; IL-6—interleukin-6; IRS-1—insulin receptor substrate-1; NF-κB—nuclear factor kappa-light-chain-enhancer of activated B cells; OXPHOS—oxidative phosphorylation; PI3K—phosphoinositide 3-kinase; PPARγ—peroxisome proliferator-activated receptor gamma; ROS—reactive oxygen species; SREBP-1c—sterol regulatory element-binding protein-1c; TNF-α—tumor necrosis factor alpha; ↑—increase in expression/activity/level; ↓—decrease in expression/activity/level. This figure was created using Servier Medical Art (available at https://smart.servier.com/) (accessed on 1 May 2025).

Diabetes and metabolic diseases pose a significant global health challenge. Their prevalence has steadily increased in recent decades due to sedentary lifestyles, poor dietary habits, and genetic predisposition [42]. These conditions are often referred to as ‘lifestyle’ or ‘civilisation’ diseases and are characterised by the dysregulation of various molecular processes in the body. It is essential to understand these underlying mechanisms in order to develop effective preventive, diagnostic, and therapeutic strategies. Figure 1 illustrates the key processes involved in diabetes and metabolic diseases, highlighting their interrelationships. These diseases are multifactorial and are influenced by a complex interplay of molecular processes, including genetic, epigenetic, and environmental factors [14]. Targeting molecular pathways involved in glucose metabolism, inflammation, lipid homeostasis, mitochondrial function, and epigenetic regulation may lead to development of novel therapeutic approaches and more personalised medicine [43]. Recent advances in omics technologies, such as transcriptomics, proteomics, and metabolomics, have enabled the identification of new biomarkers and therapeutic targets for metabolic disorders [44,45]. To effectively address the growing global burden of diabetes and related metabolic diseases, a deeper understanding of these molecular mechanisms is critical.

Hyperglycaemia triggers a cascade of biochemical changes that lead to the development of complications in subjects with DM [46]. These molecular perturbations affect multiple organ systems and lead to chronic complications. The main mechanisms of glucotoxicity contributing to diabetic complications include (i) protein glycation and advanced glycation end products (AGEs); (ii) activation of the polyol pathway; (iii) dysregulation of lipid metabolism; (iv) activation of the hexosamine biosynthetic pathway, which alters protein function through O-GlcNAcylation and contributes to insulin resistance and vascular complications [47]. Together, these pathways promote oxidative stress, chronic inflammation, and endothelial dysfunction, which are key drivers of diabetic complications.

Glycation can occur in two ways: enzymatically, through increased enzyme activity during hyperglycaemia and non-enzymatically, through the condensation of glucose and fructose aldehyde groups with protein amino groups. The non-enzymatic pathway is considered to be the main cause of glycation-induced damage to cells and tissues [48]. Extracellular and intracellular proteins, including myelin, tubulin, collagen, haemoglobin, and the heparin cofactor, undergo glycosylation, resulting in structural and functional damage [49].

Excess glucose activates the polyol pathway, where increased aldose reductase activity leads to excessive glucose conversion to sorbitol (from 1% to 8–10%) [50]. Sorbitol then accumulates in ocular tissues, Schwann cells, renal papillae, and pancreatic islets, thereby contributing to diabetic complications [47]. This accumulation disrupts the osmotic balance and cellular integrity of tissues, thereby promoting tissue damage and functional decline.

**Figure 2:**
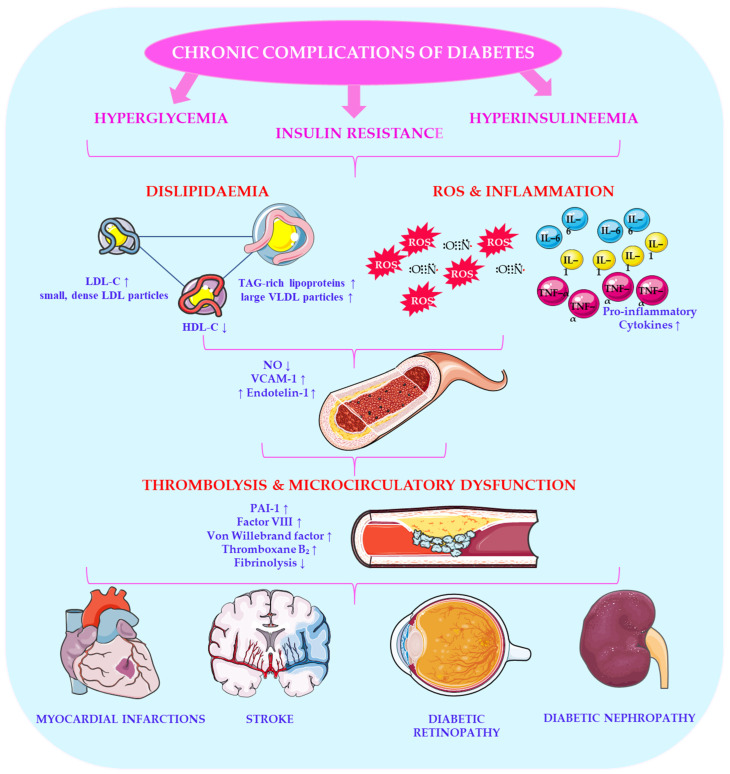
Endothelial dysfunction, dyslipidemia, and impaired hemostasis as key links in diabetic complications. Chronic complications of diabetes arise from hyperglycemia, insulin resistance, and dyslipidemia, which initiate oxidative stress, inflammation, and endothelial dysfunction. These disturbances result in impaired nitric oxide (NO) production, increased vascular permeability, and the activation of inflammatory cells and platelets. Hemostatic abnormalities, including elevated PAI-1, von Willebrand factor, and thromboxane B_2_ levels, contribute to reduced fibrinolysis and a hypercoagulable state. Together, these mechanisms promote the development of atherosclerosis and macro- and microvascular complications such as myocardial infarction, stroke, nephropathy, and diabetic retinopathy. Abbreviations: HDL-C—high-density lipoprotein cholesterol; IL-1—interleukin-1; IL-6—interleukin-6; LDL-C—low-density lipoprotein cholesterol; NO—nitric oxide; PAI-1—plasminogen activator inhibitor-1; ROS—reactive oxygen species; TAG—triacylglycerol; TNF-α—tumor necrosis factor alpha; VCAM—vascular cell adhesion molecule; VLDL—very-low-density lipoprotein; ↑—increase in expression/activity/level; ↓—decrease in expression/activity/level. This figure was created using Servier Medical Art (available at https://smart.servier.com/) (accessed on 1 May 2025).

Diabetes is associated with increased triglyceride levels and decreased high-density lipoprotein cholesterol (HDL-C) as well as often elevated low-density lipoprotein cholesterol (LDL-C) levels [51]. Dyslipidaemia significantly increases the risk of atherosclerosis and cardiovascular disease [52]. Elevated triglyceride levels are associated with insulin resistance, which contributes to atherogenic dyslipidaemia by increasing the number of small, dense LDL particles and decreasing HDL-C levels, thereby exacerbating cardiovascular risk [51]. Altered lipid profiles in diabetes often include elevated very-low-density lipoprotein cholesterol (VLDL-C) and triglyceride-rich lipoproteins, indicating abnormalities in lipid metabolism and insulin signalling pathways [51]. Figure 2 illustrates the chronic complications of diabetes, one of which is dyslipidaemia.

Hyperglycaemia, insulin resistance, and hyperinsulinaemia can lead to endothelial dysfunction, dyslipidaemia, oxidative stress, and coagulation abnormalities. These changes involve reduced fibrinolytic activity and increased plasminogen activator inhibitor-1 synthesis, both of which promote atherosclerotic plaque formation and increase cardiovascular risk [53,54]. Homeostasis and rheological disturbances play a critical role in diabetic angiopathies [55]. Endothelial dysfunction, platelet abnormalities, impaired fibrinolysis, and prothrombotic states are commonly observed in diabetes [56]. Hypercoagulation is evidenced by elevated plasma levels of thromboxane B_2_, factor VIII, von Willebrand factor, fibronectin, LDL cholesterol, and triglycerides as well as reduced fibrinolysis parameters [57]. Von Willebrand factor, synthesised by endothelial cells, mediates platelet adhesion as part of the factor VIII cofactor complex. Increased platelet activation and reduced antiplatelet activity in the vascular wall are characteristics of diabetic patients, and these factors contribute to microcirculatory dysfunction and thrombosis [58]. These haemostatic imbalances contribute to macrovascular and microvascular complications, including stroke, myocardial infarction, and diabetic retinopathy [59].

Lifestyle changes, including dietary modifications, increased physical activity, home rehabilitation using physiotherapeutic methods, and weight management, are essential for preventing and managing diabetes-related complications. It has been shown that early adoption of these interventions significantly reduces the incidence and progression of diabetes [60]. Large-scale population studies in China, Finland, and the USA have demonstrated that even modest weight loss and regular physical activity, such as walking for 30 min a day, can substantially reduce the prevalence of diabetes and prediabetes [61,62,63]. Dietary interventions play a critical role in diabetes management. Diets rich in fibre, whole grains, healthy fats (such as omega-3 fatty acids), and lean protein can help to regulate blood sugar levels and reduce insulin resistance. Studies have shown that the Mediterranean diet and low-carbohydrate diets can improve glycaemic control and reduce cardiovascular risk factors in diabetes patients [64]. Adherence and long-term outcomes are enhanced by combining nutritional strategies with behavioural support and digital health tools.

Laser therapy is a non-invasive, well-tolerated treatment representing a promising approach to diabetes management. It targets key aspects of the disease, offering novel adjunctive therapeutic options, including improved microcirculation, anti-inflammatory effects, and regulation of glucose metabolism [13,65,66,67]. Laser therapy improves tissue perfusion, alleviates symptoms of diabetic neuropathy, facilitates wound healing [68], and helps to reduce chronic inflammation, a hallmark of diabetes and its complications [69,70,71]. There is emerging evidence that laser therapy can improve insulin sensitivity and modulate mitochondrial function, thereby contributing to better glycaemic control in diabetic patients [72,73]. Its ability to influence redox balance, cytokine expression, and cellular energy metabolism establishes it as a valuable tool in the management of diabetes [74].

## 5. Therapeutic Applications of Low-Level Laser Therapy

Low-level laser therapy (LLLT), also known as photobiomodulation, uses low-intensity lasers or light-emitting diodes (LEDs) to stimulate cellular function and promote healing. Unlike high-intensity lasers, LLLT does not cut or ablate tissue, making it a non-invasive therapeutic approach [3].

LLLT primarily exerts its effects through the absorption of light by cellular chromophores, particularly cytochrome c oxidase (COX) in the mitochondrial respiratory chain. This enzyme acts as a key photoacceptor in the red and near-infrared spectral ranges, initiating cellular responses to light [75]. Covian et al. (2024) investigated the effect of the mitochondrial membrane potential (ΔΨm) on the absorbance properties of reduced COX in isolated mitochondria from rabbit hearts [76]. Using integrating sphere optical spectroscopy, the authors discovered that gradual depolarisation of ΔΨm resulted in a notable increase (up to 50%) in cytochrome aa3 absorbance, accompanied by a slight red shift. Meanwhile, cytochrome c and c1 absorbances remained unaltered. These results suggest that ΔΨm modulates the extinction coefficient of COX hemes, indicating that the mitochondrial bioenergetic state can dynamically influence the light absorption properties of the enzyme [76].

Building on this mechanism, the absorption of photons by COX triggers a cascade of photochemical and biochemical reactions. These include increased adenosine triphosphate (ATP) synthesis, modulation of reactive oxygen species (ROS) levels, and photodissociation and subsequent release of nitric oxide (NO) [4,77]. NO plays a crucial role in vasodilation, thereby enhancing blood flow and tissue oxygenation [78]. Through these interconnected pathways, LLLT supports cellular metabolism, improves microcirculation, and promotes tissue repair.

These effects are particularly relevant in the context of diabetes, where impaired mitochondrial function, oxidative stress, and endothelial dysfunction contribute significantly to the development of chronic complications [79]. Furthermore, LLLT has been demonstrated to modulate inflammatory pathways by affecting cytokine expression, which is of particular importance in chronic inflammatory conditions, such as diabetic wounds [80].

The following key laser properties and their correct combination are critical in LLLT: (i) emission wavelength; (ii) light source operating mode; (iii) emission dose; (iv) polarisation of light. Wavelengths in the visible red region (600–750 nm) are typically chosen to illuminate superficial tissues. In contrast, wavelengths in the longer range (780–950 nm) are more suitable for deeper optical penetration through tissue [77,81]. LLLT can be fulfilled by pulsed or continuous wave (CW) emission. Empirical protocols have been developed for LLLT, taking into account the light operation mode (continuous wave or pulsed), exposure time, and specific pulse parameters, such as peak power, pulse duration, and repetition rate. In the CW mode, the power and stability of the light source emission are essential [81,82]. It is also important to consider the optical properties of tissue, such as the absorption and scattering coefficients. These influence the penetration and distribution of light [83].

The concept of the radiation dose is well established in radiology, but in the context of LLLT, it refers to the amount of light energy delivered to the biological sample, which is often measured in units, such as W/cm^2^. The dose depends on several factors, including power, irradiation time, pulse duration, and repetition rate for pulsed modes. The effectiveness of LLLT depends on an optimal combination of the light dose, operating mode, and irradiation time; these parameters are the focus of experimental studies [84,85]. Light polarisation may be essential for some applications. For instance, Rubinov’s (2003) study revealed that gradient laser fields influence erythrocyte rouleaux and chromosomal aberrations, an effect that can be amplified by appropriate polarisation [86]. However, light loses its polarisation when passing through highly scattering media, such as tissue [86,87]. Therefore, the effects of polarisation are most relevant in superficial applications and when structured light delivery systems are used.

**Figure 3:**
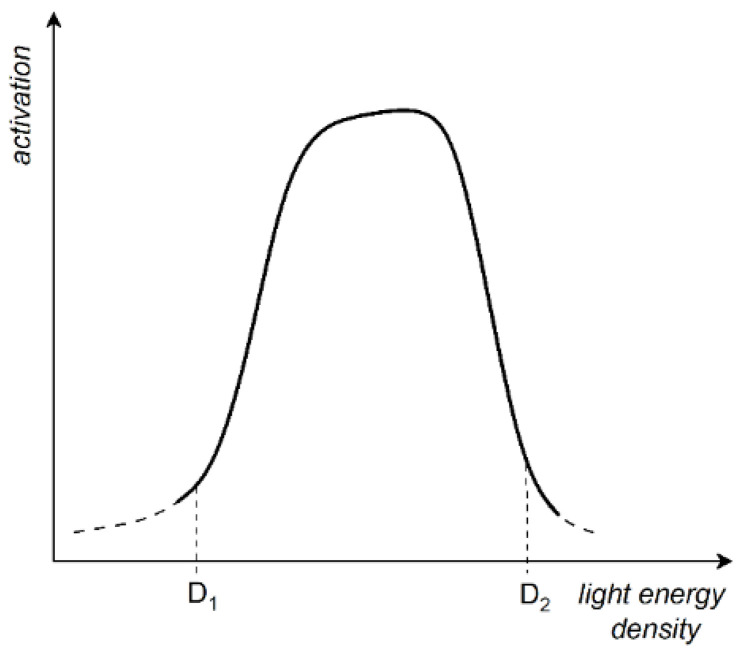
Basic Arndt–Schultz curve. The solid line represents the activation under the measured conditions, while the dashed line indicates the extrapolated activation beyond D_1_ and D_2_.

The response of biological samples to LLLT can be described by the Arndt–Schulz law (Figure 3), which states that weak light slightly accelerates metabolic activity, while stronger doses increase it up to a certain point (D1). Beyond this point, higher doses (D2) suppress the response, potentially leading to negative effects. These dose-dependent effects have been observed in several studies [88,89,90,91,92,93,94]. Obviously, the dependence shown in Figure 3 would normally depend on the other parameters of light emission, such as the mode of laser operation (pulsed or CW), wavelengths, pulse duration, and pulse repetition rate, as well as the protocols used when illuminating the samples. The energy densities that produce positive photobiological effects follow this dose–response curve. This principle is highly significant in clinical practice, as inappropriate dosing can lead not only to an absence of therapeutic effect but also to adverse outcomes. Consequently, precise dosimetry and personalised treatment planning are essential components of effective LLLT protocols.

Thanks to its non-invasive nature, LLLT is suitable for a wide range of therapeutic applications, including pain relief and promoting recovery in cases of tendinopathy [95], nerve injury [96], osteoarthritis [97], and wound healing [98]. Despite numerous studies, the full mechanism of action remains elusive. The biological effect of laser radiation is determined by the quantum and wave properties of light [99]. According to the photoresonance hypothesis, low-intensity laser irradiation selectively interacts with various acceptors, such as the respiratory enzyme cytochrome oxidase, oxygen, and haemoglobin [84,100]. These acceptors absorb light energy and undergo biophysical transformations that contribute to cellular metabolic changes. In the range of λ = 0.5–0.7 μm, these acceptors include the respiratory enzymes cytochrome oxidase and cytochrome as well as oxygen, haemoglobin, peroxide radicals, lipids, and enzymes (e.g., catalase) [101,102,103]. This wavelength range corresponds to the optical window of biological tissue, where absorption by water and haemoglobin is minimised, enabling deeper penetration [104].

The formation of a local biostimulatory effect at cellular and tissue levels is due to structural and functional reorganisation of membranes and enhanced key metabolic systems. This process is associated with the production of macroergic molecules, such as ATP, which are essential for cellular function [105,106]. Suardi et al. (2016) investigated the effect of visible laser light on the ATP levels and viability of anaemic red blood cells using lasers that emitted light at wavelengths of 460 nm and 532 nm [107]. The analysis of ATP levels in anaemic and normal erythrocytes before and after the exposure to lasers of different durations (30, 40, 50, and 60 s) showed that ATP levels increased specifically within the anaemic erythrocyte population following the irradiation. Additionally, mitotic activity and the surface adhesion properties of cells normalised, activating local repair processes and stimulating the immune system [108]. In diabetic patients, where red blood cell deformability and ATP synthesis are impaired, such findings may indicate the therapeutic potential of LLLT in improving microcirculation and oxygen delivery. This suggests that LLLT could be used alongside other treatments to help manage diabetic complications, particularly peripheral vascular disease.

In the pathogenetic mechanism of action of low-level laser therapy on biological tissues, the main role is played by the absorption of light by epidermal macrophages (also known as Langerhans cells) [109], which triggers a reaction in the microcirculatory system (initially in the arteries, then the veins and lymphatic vessels) in the area exposed to light, which later becomes widespread [110]. Capillary blood flow is activated through the opening of previously non-functioning capillaries, and repeated exposure results in an increase in the capillary network or neovascularisation, as observed by Lambert et al. (2013) [111]. Such effects are particularly important in the healing of diabetic wounds, where impaired angiogenesis and reduced capillary density delay tissue repair [112]. Furthermore, LLLT has been shown to increase the expression of vascular endothelial growth factor (VEGF), which is a key mediator of angiogenesis [113].

Numerous studies suggest that fluences in the range of 3 to 10 J/cm^2^ optimally stimulate metabolic activity at the cellular level [68,85,114]. Biostimulation occurs at an open wound in the range of energy densities from 0.5 to 1 J/cm^2^ and at a target through the overlying skin in the range from 2 to 4 J/cm^2^ [94]. Other studies [115,116] suggest that doses of 4 J/cm^2^ should be used for superficial targets, within a range of 1–10 J/cm^2^. Doses of 4 J/cm^2^ are generally effective for superficial targets, while deeper targets may require doses in the range of 10 to 50 J/cm^2^ [117]. This wide therapeutic window highlights the necessity of adjusting LLLT protocols individually, particularly for chronic diseases, such as diabetes, where tissue response may vary significantly. In clinical settings, precise dosimetry is required as well as consideration of patient-specific factors, such as skin pigmentation, vascularisation, and the chronicity of the condition being treated [80].

Although significant heterogeneity exists in the reported PBMT/LLLT protocols, most studies converge within specific therapeutic windows, particularly regarding wavelength (600–700 nm for superficial targets and 780–950 nm for deeper tissues) and fluence (generally 1–10 J/cm^2^ for superficial applications and up to 50 J/cm^2^ for deeper structures). The values presented in Table 1 should not be interpreted as strict clinical recommendations but rather as guiding ranges derived from the most frequently reported parameters in experimental and clinical studies. Further standardised, large-scale clinical trials are necessary to establish evidence-based consensus protocols for diabetes-related indications.

Other studies [118,119] investigated the cellular and molecular mechanisms underlying photobiomodulation, including the activation of cellular signalling pathways and the modulation of gene expression and mitochondrial function. The therapeutic effects of low-intensity laser irradiation with beam powers ranging from 1 to 100 mW are due to the reversible modification of blood components and enzymes that integrate into the body’s normal bioenergetic structure. Targeting specific frequencies significantly enhances the therapeutic effects of laser therapy, as demonstrated by Kim and Jeong (2014) [120], Farivar et al. (2014) [4], and Reis et al. (2022) [121]. It is thought that these frequency-dependent effects arise from resonance interactions between photon energy and molecular vibrational states, which selectively activate biochemical pathways [102].

There is emerging evidence suggesting that LLLT could be a valuable addition to diabetes management strategies, improving wound healing, reducing neuropathic pain, and enhancing tissue regeneration. In particular, its capacity to regulate oxidative stress and inflammatory mediators provides a promising approach to mitigating the development of diabetic complications, such as peripheral neuropathy and chronic ulcers [98,112].

## 6. Types of Biomodulation Most Commonly Used in LLLT in Medical Practice

Laser devices generate light that is qualitatively different from the light of conventional light sources because the mechanism of its generation-stimulated emission differs principally from the spontaneous emission of conventional sources. The main advantages of laser light lie in its extremely high monochromaticity, i.e., the ability to concentrate radiation within a record narrow spectral range (Δλ) down to picometers (10–12 m) and a possibility to get radiation in various spectral ranges required for the experiment. Monochromaticity is directly related to the coherence of laser light waves generated at identical frequencies and phases. An important quality of laser radiation is the ability to generate directed beams of light with a very low divergence of 10^−4^ radians, which is hundreds of times smaller than the divergence of traditional light sources. This allows energy to be concentrated on small surfaces. Different types of lasers can produce electromagnetic radiation with a very small beam divergence angle, spanning ultraviolet to infrared wavelengths [4,122]. Another valuable property is the ability to operate in various time modes, from steady-state to pulsed, with pulses of varying durations down to fs (i.e., 10–15 s), and in pulse repetition mode, from single pulses to higher rates reaching the MHz range.

For many applications, radiation power and energy are important, and the range over which these parameters are available is also unique—from a single-photon regime to energies heating plasma to millions of degrees and triggering thermonuclear reactions. The light interaction with material objects is highly dependent on the pulse duration and energy characteristics together with the wavelength of light. The latter properties open up unique opportunities to induce various changes into interacting samples, including biological ones [104,105].

Lasers typically generate light in active media capable of amplifying light in optical resonators. There are several classifications of lasers based on the type of active medium (gas, solid-state, liquid, and semiconductor), spectral range, operating mode, pulse duration and frequency, and intended application. Various types of lasers are used in biology and medicine, from low-intensity ones for therapeutic purposes to high-intensity lasers used in surgical and ablative procedures. Each type of laser therapy may offer unique advantages and applications to meet various clinical needs and treatment objectives [104,105].

The first medical applications of laser light were reported in the 1960s, shortly after the laser was invented by Maiman in 1960. Since then, its therapeutic potential has been systematically explored in such fields as dermatology, ophthalmology, oncology, and rehabilitation medicine [104,105]. The development of more precise and tunable laser systems over time has enabled targeted interventions with minimal collateral damage, thus expanding the scope of laser therapy in both acute and chronic conditions [123]. Understanding the characteristics and capabilities of different laser modalities enables healthcare professionals to utilise laser therapy effectively as a valuable tool in managing various medical conditions.

Laser therapy involves the use of laser light for therapeutic purposes [123,124]. The different types of laser therapy are distinguished by their wavelengths, power densities, and treatment targets. The following summary outlines each type of therapy and its applications. This information can be applied to the treatment of diabetes [16,17]. It is important to recognise that diabetes is a multifactorial disease affecting multiple organs and systems and that laser therapy applications must be tailored to the specific needs of diabetic patients. Furthermore, recent evidence indicates that PBMT can enhance microcirculation and modulate oxidative stress, thereby accelerating wound healing in diabetic patients [73,125]. In addition to wound healing, PBMT has shown promise in alleviating symptoms of diabetic neuropathy by modulating nociceptive pathways and reducing inflammatory cytokine levels [126,127]. These findings highlight the potential of laser therapy as a complementary approach in comprehensive diabetes management.

Table 2 shows the types of biomodulation most commonly used in LLLT in medical practice.

### 6.1. Low-Level Laser Therapy (LLLT)

LLLT is considered a non-specific therapeutic factor that aims to photochemically activate biochemical processes within biological tissues. This modulation occurs without inducing thermal damage or other structural changes. Essentially, it acts as an optical catalyst for cellular biochemical activity, thereby enhancing the physiological functions of the entire organism, including the neuroendocrine, endocrine, immune, and vascular systems [3,128]. This modulation occurs without inducing thermal damage and other structure changes, which is remarkable and distinguishes LLLT from high-intensity laser applications used in surgical contexts. Consequently, LLLT has a wide range of applications in rehabilitative medicine. As the research into this method progresses and the experience in treating various diseases increases, the use of LLLT is expanding.

LLLT has shown significant potential in treating chronic pain [95,129], promoting tissue repair [3,130], and modulating inflammation [131]. It is a treatment in which the light energy absorbed and scattered by the body does not increase its temperature by more than 1 °C. Exposure to LLLT (monochromatic, polarised, coherent light with a radiant power of several milliwatts concentrated within a few microns) results in optical effects typically observed when light passes through an inhomogeneous medium in a biological environment [132]. These optical interactions include scattering, refraction, and selective absorption, influencing the depth and distribution of photobiological effects.

LLLT uses low-intensity laser light, typically ranging from one to one thousand milliwatts. It is commonly used for its tissue-healing, pain-relieving, and anti-inflammatory properties [133,134]. It is frequently used to treat musculoskeletal conditions, promote wound healing, and address dermatological issues [3,95]. It has also been shown to be effective in preventing oral mucositis in stem cell transplant recipients undergoing chemotherapy [135]. In the treatment of musculoskeletal conditions, LLLT has shown promise in providing short-term pain relief for such conditions as rheumatoid arthritis, osteoarthritis, chronic low back pain, neck pain, tendinopathy, chronic joint disease, and oncology-related pain [95,128].

However, the effectiveness of LLLT in dentistry and wound healing remains controversial. This variability may be attributed to differences in treatment protocols, including wavelength selection, energy density, and irradiation time, which are not yet standardised across clinical settings [80]. Nevertheless, evidence suggests that the effects of LLLT are related to specific wavelengths of laser light that primarily target the respiratory enzyme cytochrome c oxidase, which plays a role in the electron transport chain within mitochondria [136]. This interaction stimulates and enhances cell function, promotes wound repair, and reduces inflammation. Furthermore, cytochrome c oxidase activation results in increased ATP synthesis, enhanced cellular metabolism, and modulation of transcription factors that are involved in tissue regeneration [101,102].

Cold laser therapy, also known as LLLT, is a non-invasive medical treatment that uses low-level lasers or light-emitting diodes (LEDs) to stimulate cell function and promote tissue healing [137,138]. Widely used in sports medicine and dermatology, it is particularly effective in reducing pain, controlling inflammation, and improving wound healing [3,139]. It promotes tissue repair, reduces inflammation, and accelerates wound healing [140]. Cold laser therapy is also used to treat skin conditions, such as acne, scars, and psoriasis [3,139]. Unlike HILT, cold laser therapy operates at lower power outputs (typically < 500 mW), making it safer for superficial applications and sensitive areas, such as the face or mucosal surfaces [104]. The effectiveness of cold laser therapy depends on such parameters as wavelength, fluence, and treatment duration, all of which must be carefully adjusted according to the clinical indication.

### 6.2. High-Intensity Laser Therapy (HILT)

As reported by Starzec-Proserpio et al. (2022) [141], HILT is becoming increasingly popular for treating chronic musculoskeletal pain, including such conditions as vulvodynia and fibromyalgia. The therapy aims to alleviate pain and enhance functionality in chronic inflammatory and degenerative conditions [142]. The mechanism of action of HILT is based on biostimulation [143]. High-intensity laser radiation provides cells with additional energy, thereby affecting their metabolism and increasing ATP production in the mitochondria [144]. This promotes cellular repair and regeneration as well as anti-inflammatory responses, making HILT effective in rehabilitative medicine. Due to its ability to penetrate deep tissue (by several centimetres), HILT is particularly well-suited to treating large joints, deep muscle layers, and neuropathic pain syndromes [90]. The therapy typically uses wavelengths in the near-infrared range (e.g., 1064 nm), enabling effective energy delivery to subcutaneous structures.

### 6.3. Pulsed Laser Therapy

In pulsed laser therapy, laser light is delivered in short, repeated bursts rather than continuously [145,146]. This modulation enables precise control of the treatment parameters, thereby enhancing the cellular response and tissue repair. Pulsed laser therapy is used in several medical fields, including orthopaedics, dermatology, and sports medicine. The mechanism of action involves delivering energy in short bursts (in the nanosecond to microsecond range), which allows precise tissue targeting while minimising thermal damage. It is particularly effective in promoting angiogenesis, modulating inflammatory processes, and stimulating ATP production [133,147,148]. Recent studies also suggest that pulsed emission can reduce the risk of phototoxicity and improve patient comfort during treatment, particularly in long-term protocols [123,124]. Furthermore, the ability to adjust pulse parameters (e.g., frequency, peak power, and duty cycle) enables clinicians to customise therapy for each patient and tissue type.

### 6.4. Superpulsed Laser Therapy

Superpulsed laser therapy uses short pulses of high-intensity laser light to deliver energy quickly, reducing treatment times. It offers such benefits as deeper tissue penetration, increased precision, and greater patient comfort. It is commonly used to treat musculoskeletal conditions, promote wound healing, and manage pain [143,149]. The superpulsed mode enables high peak power to be delivered with minimal thermal accumulation, making it suitable for treating inflamed or sensitive tissues without causing additional irritation [144]. This modality is particularly effective in stimulating microcirculation and accelerating tissue regeneration, especially in chronic injuries and post-operative recovery.

### 6.5. Hot Laser Therapy

Hot laser therapy uses high-intensity laser light to generate heat within tissues, resulting in thermal effects, such as increased blood flow, tissue relaxation, and pain relief [150]. It is primarily used in physiotherapy and rehabilitation to treat conditions that require deep tissue heating, such as muscle spasms, joint stiffness, and chronic inflammatory conditions [139,151]. The thermal response induced by hot laser therapy also facilitates collagen remodelling and enhances tissue elasticity, which is beneficial in treating fibrotic conditions and post-traumatic stiffness [90]. However, careful control of dosage and exposure time is essential to avoid overheating and tissue damage.

### 6.6. Clinical Relevance and Integration

Each type of laser therapy has unique benefits and applications that are tailored to specific clinical needs and treatment goals. Understanding the characteristics and capabilities of different laser modalities enables practitioners to optimise laser therapy for such conditions as musculoskeletal disorders, wound healing, pain management, and inflammatory diseases (Table 2). Combining different laser modalities, such as alternating superpulsed and cold laser therapy, may enhance therapeutic outcomes by leveraging both photothermal and photochemical mechanisms [133].

There is a growing body of evidence supporting the use of laser therapy in treating diabetic complications, such as neuropathy, impaired wound healing, and microvascular dysfunction [71,152,153]. Laser therapy has particularly demonstrated its efficacy in improving endothelial function, reducing oxidative stress, and enhancing angiogenesis in diabetic tissues. These are all critical factors in restoring vascular integrity and promoting tissue repair [125].

## 7. Therapeutic Potential of Low-Level Laser Therapy in Diabetes Management

In modern rehabilitation medicine, low-energy laser radiation is widely used. The key technical parameters of laser therapy devices, such as wavelength, output power, and pulse repetition frequency, are closely linked to the depth of tissue penetration, light energy distribution in the sample, and biological effects. In the rehabilitation of diabetic patients, the average power of LLLT is usually less than 100 mW on the wavelengths in the red and infrared (IR) part of the spectrum. The irradiation time must be carefully adjusted to achieve the desired dose, taking into account the size and location of the target area. The therapeutic efficacy of LLLT is largely attributed to its profound impact on components of the mitochondrial respiratory chain, which initiates a cascade of biological responses with far-reaching implications for diabetes treatment. Several studies [83,102] have demonstrated that these mechanisms explain how low-intensity laser light activates mitochondrial respiratory components [154]. This interaction is wavelength-dependent, with optimal biological responses observed in the red to near-infrared spectrum (600–950 nm), corresponding to the absorption peaks of cytochrome c oxidase [125].

**Figure 4:**
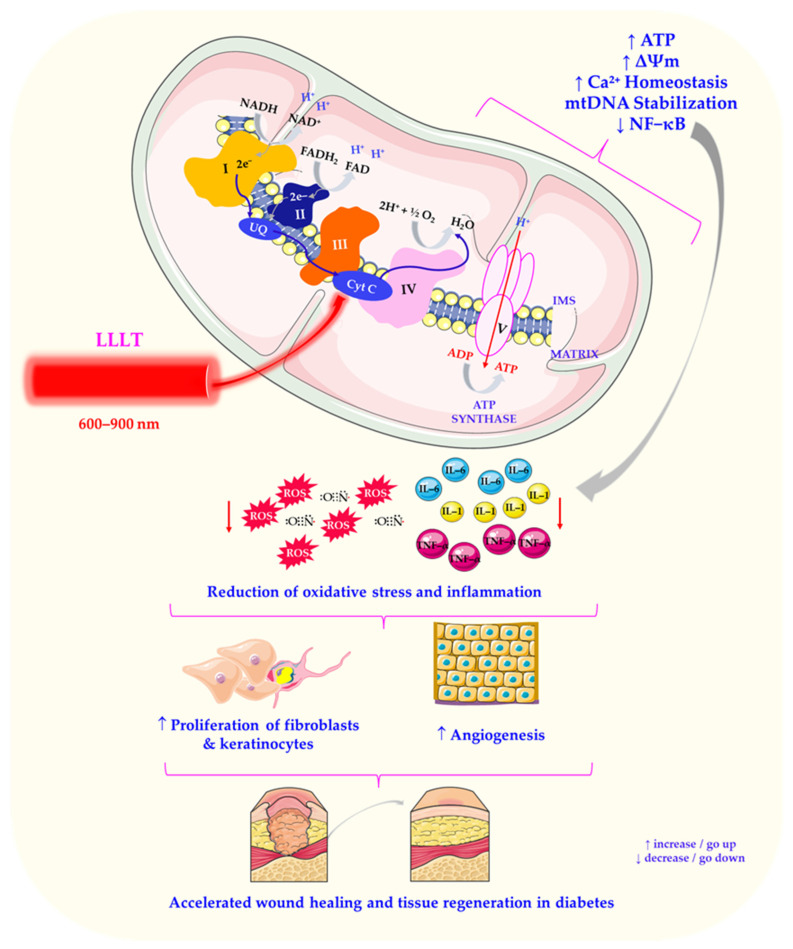
Therapeutic effects of low-level laser therapy on mitochondrial function and diabetic tissue repair. Low-level laser therapy (LLLT) activates cytochrome c oxidase (complex IV), enhancing electron transport, ATP synthesis, and mitochondrial stability. Increased cellular bioenergetics and inhibition of the NF-κB pathway reduce inflammation and oxidative stress, promoting fibroblast proliferation, angiogenesis, and tissue regeneration in patients with diabetes. Abbreviations: ADP—adenosine diphosphate; ATP—adenosine triphosphate; Cyt C—cytochrome c; FAD—flavin adenine dinucleotide; FADH_2_—reduced flavin adenine dinucleotide; IL-1—interleukin-1; IL-6—interleukin-6; IMS—intermembrane space; LLLT—low-level laser therapy; MATRIX—mitochondrial Matrix; mtDNA—mitochondrial DNA; NAD^+^—nicotinamide adenine dinucleotide (oxidized form); NADH—reduced nicotinamide adenine dinucleotide; NF-κB—nuclear factor kappa-light-chain-enhancer of activated B; NO—nitric oxide; ROS—reactive oxygen species; TNF-α—tumor necrosis factor alpha; UQ—ubiquinone (coenzyme Q); ΔΨm—mitochondrial membrane potential. I–V denote mitochondrial respiratory chain complexes: I—Complex I (NADH dehydrogenase), II—Complex II (succinate dehydrogenase), III—Complex III (cytochrome bc1 complex), IV—Complex IV (cytochrome c oxidase), V—Complex V (ATP synthase). Arrows indicate direction of processes and changes: ↑ increase or upregulation; ↓ decrease or downregulation; red arrows indicate stimulation by LLLT; dashed arrows indicate indirect or secondary effects. This figure was created using Servier Medical Art (available at https://smart.servier.com/) (accessed on 1 May 2025).

Mitochondria play a pivotal role in cellular metabolism, energy production, and signalling pathways, with the electron transport chain (ETC) at the heart of their functionality. The ETC consists of protein complexes embedded in the inner mitochondrial membrane, which coordinate electron transfer and ATP synthesis [155]. The transfer of electrons through these complexes stimulates ATP production, which is the primary energy currency of the cell [125,156,157]. In order to understand how LLLT modulates these processes, it is crucial to identify its primary molecular targets (Figure 4).

One of the main mechanisms by which low-intensity laser light affects mitochondrial function is the activation of COX, which contains haem and copper centres that absorb light in the near-infrared region [125]. COX is the terminal component of the mitochondrial respiratory chain [158] and is also functionally associated with the endoplasmic reticulum and plasma membrane [159]. As demonstrated by Lebiedzinska et al. (2009), the dynamic structural and functional connections between mitochondria, the plasma membrane, and other organelles are crucial for intracellular phospholipid transport, calcium homeostasis, and cyclic AMP regulation [160]. These processes govern fundamental cellular functions, such as motility, contraction, secretion, growth, proliferation, and apoptosis [125]. Disruption to these pathways is commonly observed in diabetic tissues, further supporting the rationale for mitochondrial-targeted therapies such as LLLT.

Cytochrome c oxidase, also known as complex IV, is the primary site of electron transfer to molecular oxygen, facilitating ATP production via oxidative phosphorylation [161]. Studies have shown that exposure to low-intensity laser light increases cytochrome c oxidase activity, resulting in enhanced electron flow and ATP synthesis [162,163]. This ATP upregulation is particularly important from a clinical perspective, as it supports the cellular processes required for repair, regeneration, and homeostasis—especially in the context of diabetes-related complications, such as impaired wound healing and tissue ischaemia. Furthermore, increased ATP availability improves fibroblast migration and keratinocyte proliferation, both of which are essential for re-epithelialisation in chronic diabetic wounds [68].

Beyond ATP synthesis, LLLT has been shown to modulate mitochondrial membrane potential [164], calcium homeostasis [165], and mitochondrial DNA integrity, highlighting its multifaceted impact on mitochondrial function. By enhancing mitochondrial bioenergetics and signalling, LLLT optimises cellular metabolism, promotes proliferation, and improves tissue resilience [166]. These effects translate into improved wound repair and reduced inflammation in diabetic patients, thus linking mitochondrial activation directly to clinically relevant outcomes.

Furthermore, the activation of mitochondria by near-infrared laser light has been shown to have benefits beyond wound healing. For example, Yang et al. (2023) found that laser treatment reduced oxidative stress and repaired tissue damage in the corpus cavernosum of diabetic rats, thereby improving erectile dysfunction [167]. These findings highlight the potential of LLLT in clinical practice, although further validation in human subjects is required. Such applications suggest that LLLT could be beneficial in repairing peripheral tissue and addressing systemic complications of diabetes, including vascular and neurological dysfunctions.

Additionally, LLLT activates mitochondrial respiratory components, triggering a cascade of cellular signalling pathways with significant physiological effects [102]. Mitochondria are known to produce ROS, which contribute to oxidative stress but also serve as signalling molecules in cellular adaptation [157]. Laser irradiation generates moderate ROS levels, initiating protective responses that prevent excessive inflammation [80]. One such pathway involves ROS-mediated activation of redox-sensitive transcription factors, including nuclear factor-κB (NF-κB) [168] and hypoxia-inducible factor 1-alpha (HIF-1α) [169], which coordinate stress responses and regulate wound healing.

Beyond mitochondrial activation and ROS modulation, increasing evidence indicates that photobiomodulation influences intracellular signalling pathways involved in cell cycle regulation, particularly the mitogen-activated protein kinase (MAPK) cascade [91,92]. Activation of MAPK pathways, including extracellular signal-regulated kinase (ERK1/2), appears to play a central role in mediating PBMT-induced cellular proliferation, differentiation, and survival. Low-level laser irradiation has been shown to induce transient ROS generation, which acts as a secondary messenger triggering MAPK/ERK phosphorylation. This activation subsequently regulates transcription factors controlling cyclin expression and cell cycle progression [80,144]. In non-destructive energy ranges, ERK activation is generally associated with pro-survival and regenerative responses, including enhanced fibroblast proliferation, endothelial cell migration, and tissue repair. However, the magnitude and duration of MAPK signalling appear to be dose-dependent, further supporting the biphasic dose–response phenomenon described for PBMT [83,102]. These findings suggest that MAPK/ERK pathways constitute an important bridge between primary photochemical events at the mitochondrial level and downstream transcriptional regulation, linking laser-induced redox modulation with functional cellular outcomes [80,144].

Importantly, diabetes has been linked to impaired wound healing and an extended inflammatory phase [112,170]. Elevated levels of pro-inflammatory cytokines, such as interleukin (IL)-1β, tumour necrosis factor (TNF)-α, and IL-6, contribute to delayed tissue repair. In a study on human skin fibroblast cell lines, Sekhejane et al. (2011) demonstrated that low-intensity laser irradiation reduced pro-inflammatory cytokine levels in diabetic and hypoxic conditions [168]. Phototherapy accelerated wound closure and increased cell proliferation by normalising cellular function and reducing cytokine expression and NF-κB translocation in a time-dependent manner [171]. These findings support the use of LLLT as a non-pharmacological strategy to modulate inflammation and promote tissue regeneration in diabetic patients.

The activation of mitochondrial respiratory chain components is a key mechanism underlying the therapeutic effects of low-intensity laser light in diabetes management [73]. By harnessing the power of light to stimulate mitochondrial function, LLLT offers a non-invasive, targeted approach to improving cellular metabolism, promoting tissue repair and mitigating diabetes-related complications. Due to its non-invasive nature, minimal side effects, and potential for multiple benefits, laser therapy is poised to become a valuable addition to diabetes management strategies. Understanding the impact of laser therapy on various aspects of diabetes pathophysiology could inform the development of innovative approaches to improving outcomes for people living with this chronic disease [17,21]. In particular, LLLT may complement pharmacological interventions by enhancing tissue responsiveness and accelerating recovery in patients with poor glycaemic control.

However, it is important to note that, despite the promising evidence, the mechanisms by which LLLT modulates mitochondrial activity and systemic responses in diabetic patients are still under investigation. Further studies are needed to establish the optimal treatment parameters (e.g., wavelength, dose, duration, and frequency of irradiation) and to validate the long-term safety and efficacy of the treatment in clinical practice. Furthermore, when designing personalised laser therapy protocols, it is crucial to consider patient-specific factors, such as skin pigmentation, vascular density, and the stage of diabetic complications [80].

Although many biological effects of PBMT/LLLT are shared across different forms of diabetes, their relevance may vary depending on the underlying pathophysiology (Table 3). Mechanisms such as improved microcirculation through nitric oxide release, reduction in oxidative stress, mitochondrial activation, and enhanced ATP synthesis appear to be beneficial in all types of diabetes, as they address common downstream consequences of chronic hyperglycaemia.

In T1DM, where autoimmune-mediated β-cell destruction and chronic inflammation predominate, the immunomodulatory and anti-inflammatory effects of PBMT—including NF-κB modulation, cytokine reduction, and macrophage phenotype regulation—may be particularly relevant. Experimental data suggesting potential preservation of β-cell viability further support this perspective. In contrast, in T2DM, characterised primarily by insulin resistance and metabolic dysregulation, PBMT may exert more pronounced translational benefits through modulation of insulin signalling pathways (e.g., PI3K/Akt), improvement in mitochondrial function, enhancement of GLUT4-mediated glucose uptake, and reduction in chronic low-grade inflammation associated with adipose tissue dysfunction. This differentiation highlights the potential for tailoring PBMT protocols according to diabetes phenotype, thereby increasing its precision and clinical applicability.

From 2004 to 2024, the PubMed database documented over 1900 publications focusing on laser therapy and diabetes. However, over the last five years (2020–2025), this figure has increased significantly, reflecting the growing interest in this area of research. This highlights the increasing recognition of photobiomodulation as a potential addition to diabetes management and the need for narrative reviews to summarise clinical outcomes. Despite the expanding body of literature, there is still a lack of standardised clinical guidelines, which highlights the importance of interdisciplinary collaboration between researchers, clinicians, and biomedical engineers.

Table 4 is based on data from biomedical and scientific articles in medicine and biology from the last five years. It focuses on changes in metabolic and clinical parameters following laser therapy in diabetic patients. These parameters include improved wound closure rates, reduced pro-inflammatory cytokine levels, enhanced microcirculation and modulated oxidative stress markers, each of which contributes to better clinical outcomes in diabetes care.

## 8. Mechanisms of Action of Laser Therapy

### 8.1. Photobiomodulation and Mitochondrial Activation

One of the fundamental mechanisms by which LLLT exerts its therapeutic effects is the activation of components of the mitochondrial respiratory chain, particularly cytochrome c oxidase. This photoreceptor absorbs light in the red-to-near-infrared spectrum, resulting in enhanced electron transport and increased oxygen consumption, ultimately leading to elevated ATP production [4,163]. As the cell’s universal energy currency, ATP supports vital processes, such as repair, regeneration, and tissue homeostasis, which are often impaired in diabetes [157]. This mechanism is particularly important in diabetic tissues, where impaired wound healing and chronic inflammation are often caused by mitochondrial dysfunction and reduced ATP synthesis [112].

In clinical practice, the infrared spectral range is most widely used due to its superior tissue penetration and alignment with mitochondrial absorption peaks. Biological structures possess their own oscillatory frequencies, and resonance phenomena may occur when these coincide with the external pulsed laser light frequency. This restores the ‘electromagnetic frame’ of the cell or tissue and promotes structural repair [191]. Such resonance-based interactions may enhance cellular coherence and synchrony, thereby improving intercellular communication and tissue-level responses.

External electromagnetic light influences with frequencies between 0.01 and 0.5 Hz, which are close to the frequencies of living systems, exert particularly strong biological effects. Modulation frequencies between 1 and 10,000 Hz are typically applied to specific organs and tissues, whereas frequencies ranging from 0.1 to 100 Hz are commonly employed for acupuncture points [192,193]. Therefore, careful selection of wavelength and modulation frequency of pulses is essential in tailoring therapeutic interventions for diabetic patients. There is emerging evidence that frequency-specific photobiomodulation can affect gene expression, cytokine release, and mitochondrial dynamics differently, offering a way to create personalised laser therapy protocols [80,144,161].

### 8.2. Effects on Oxidative Stress, Nitric Oxide Release, and Microcirculation

In addition to activating mitochondria, LLLT significantly affects oxidative stress and nitric oxide (NO) dynamics. The absorption of light by cytochrome c oxidase promotes the photodissociation of NO bound to the enzyme, thereby increasing the availability of free NO [4,194]. NO is a multifunctional signalling molecule playing a pivotal role in vascular homeostasis, neurotransmission, and immune regulation [78]. By enhancing NO release, LLLT supports vasodilation, improves microcirculation, and increases tissue oxygenation—mechanisms that are particularly relevant in the context of diabetic complications, such as peripheral ischaemia and diabetic foot ulcers [144,195,196]. These vascular effects are particularly important for diabetic patients, in whom endothelial dysfunction and impaired perfusion can lead to delayed wound healing and an increased risk of tissue necrosis [112].

Experimental evidence highlights the importance of nitric oxide in these processes. Karu et al. (2005) [194] demonstrated that specific wavelengths (619–820 nm) can enhance the attachment of HeLa cells through cytochrome c oxidase-mediated signalling. However, pre-irradiation with NO donors modified this effect, suggesting that NO binding to the enzyme plays a regulatory role [194]. Similarly, Atum et al. (2022) showed that preconditioning with PBMT reduced doxorubicin-induced cardiotoxicity in human cardiomyocytes by lowering oxidative stress levels and restoring endothelial nitric oxide synthase (eNOS) activity [197]. Restoration of eNOS function is critical because diabetes is known to suppress eNOS expression and activity, resulting in reduced NO bioavailability and vascular complications [170].

**Figure 5:**
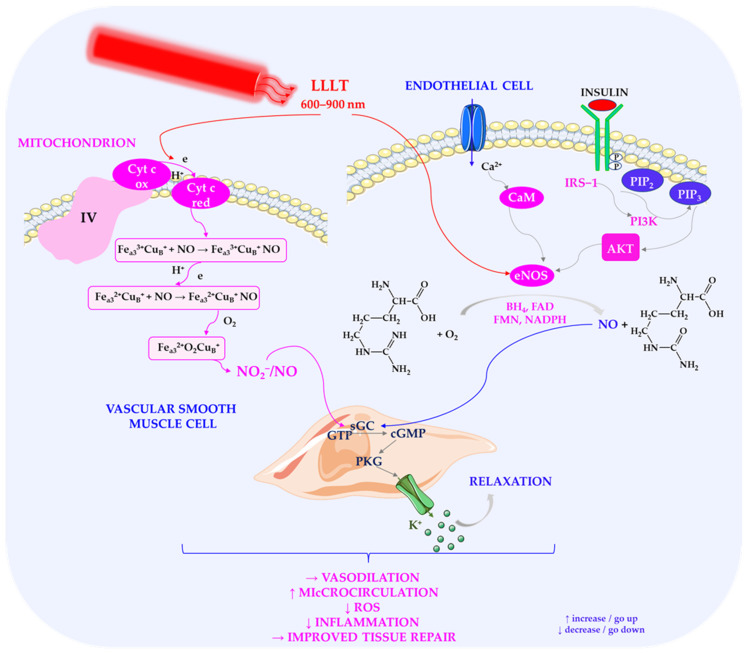
Effects of photobiomodulation on nitric oxide signaling and microcirculation in diabetes. Photobiomodulation (LLLT) activates mitochondrial cytochrome c oxidase, leading to the photodissociation of nitric oxide (NO) and enhanced mitochondrial respiration. This process increases NO bioavailability and restores endothelial nitric oxide synthase (eNOS) activity, which is often impaired in diabetes. The resulting improvement in endothelial function promotes vasodilation, enhances microcirculatory perfusion, and reduces oxidative stress and inflammation. Collectively, these effects improve tissue oxygenation, support wound healing, and protect against diabetic vascular complications. Abbreviations: AKT—protein kinase B (PKB); BH_4_—tetrahydrobiopterin; CaM—calmodulin; cGMP—cyclic guanosine monophosphate; Cyt c (ox)—cytochrome c (oxidized form); Cyt c (red)—cytochrome c (reduced form); eNOS—endothelial nitric oxide synthase; FAD—flavin adenine dinucleotide; FMN—flavin mononucleotide; GTP—guanosine triphosphate; IRS-1—insulin receptor substrate-1; LLLT—low-level laser therapy; NADPH—nicotinamide adenine dinucleotide phosphate (reduced form); NO—nitric oxide; PI3K—phosphoinositide 3-kinase; PIP_2_—phosphatidylinositol 4,5-bisphosphate; PIP_3_—phosphatidylinositol 3,4,5-trisphosphate; PKG—protein kinase G; sGC—soluble guanylyl cyclase; ROS—reactive oxygen species; IV—Complex IV (cytochrome c oxidase). Each arrow type: red arrow—light/laser effect; black arrow—electron or molecule flow; blue arrow—NO signaling; gray arrow—downstream effects/secondary pathways; pink colour—mitochondrial proteins; purple—signaling molecules/cofactors. This figure was created using Servier Medical Art (available at https://smart.servier.com/) (accessed on 1 May 2025).

Figure 5 presents the effects of photobiomodulation on nitric oxide signalling and microcirculation in diabetes.

Taken together, these findings suggest that modulation of the NO pathway is crucial for the beneficial vascular and cytoprotective effects of PBMT. In addition to promoting vasodilation, NO modulates leukocyte adhesion and platelet aggregation, potentially reducing chronic inflammation and thrombosis risk in diabetic patients [80]. Therefore, targeting NO signalling through LLLT is a promising strategy for improving microvascular health and mitigating the progression of diabetes-related tissue damage.

### 8.3. Anti-Inflammatory and Neuroprotective Mechanisms

Chronic inflammation is a hallmark of diabetes, contributing to insulin resistance, beta-cell dysfunction, and vascular complications [198]. Laser therapy has emerged as a promising strategy for modulating inflammatory responses. LLLT can induce ROS generation in physiological conditions; however, in stressed or diseased cells, it reduces ROS accumulation while enhancing antioxidant defences [80]. Notably, PBMT modulates the activity of nuclear factor kappa B (NF-κB): it activates NF-κB in resting cells but inhibits its pro-inflammatory activity in activated cells, thereby reducing cytokine release [199,200]. This dual regulatory effect enables PBMT to maintain immune vigilance while preventing chronic inflammation, which is particularly beneficial in the context of tissue damage associated with diabetes.

In addition to modulating NF-κB, PBMT influences the polarisation of macrophages by reducing the expression of markers of the M1 phenotype, thereby shifting immune responses towards tissue repair. It also reduces the production of prostaglandins and reactive nitrogen species, as demonstrated in multiple preclinical models [3,84]. This shift from a pro-inflammatory to a reparative macrophage phenotype improves wound healing and reduces fibrosis in diabetic tissues [170]. This immunomodulatory effect is particularly important for diabetic patients, as chronic low-grade inflammation can lead to insulin resistance and vascular complications. Furthermore, PBMT has been shown to reduce levels of inflammatory cytokines, such as IL-6, TNF-α, and MCP-1, which are increased in patients with type 1 and type 2 diabetes [22]. Additionally, laser therapy has been shown to impact mucosal immunity by modulating dendritic cell activity and enhancing regulatory T-cell responses. This promotes immune tolerance and reduces autoimmune activation [74,201].

**Figure 6:**
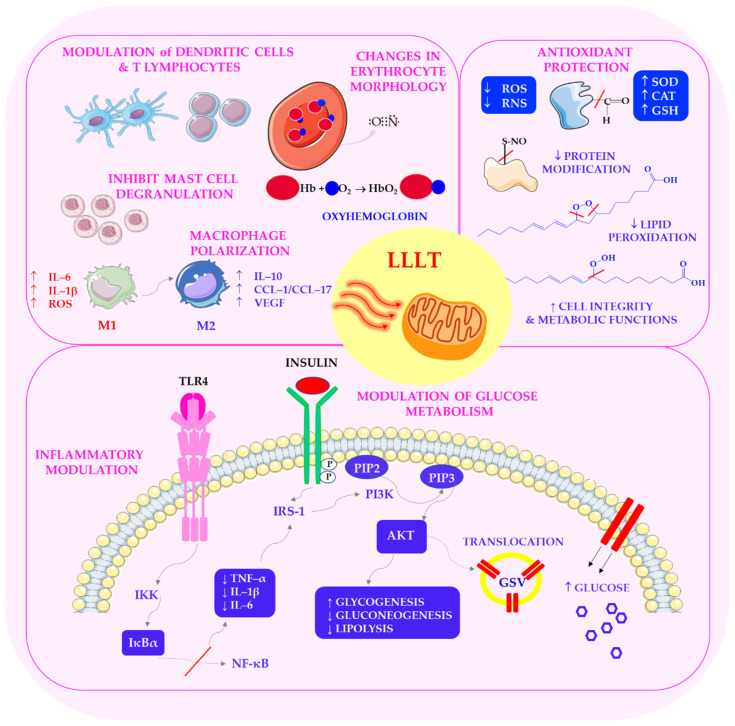
Photobiomodulation in diabetes: anti-inflammatory, antioxidant, and metabolic regulatory mechanisms. Photobiomodulation modulates inflammatory and oxidative stress responses in diabetes by regulating NF-κB activity, reducing pro-inflammatory cytokines, and shifting macrophage polarization from the M1 to the M2 phenotype. PBMT enhances mitochondrial biogenesis and activates intracellular signaling cascades involved in insulin regulation, including the PI3K/Akt pathway. It improves endothelial function, increases NO and ATP production, and activates repair pathways (VEGF, FGF), promoting angiogenesis, tissue regeneration, and diabetic wound healing. Additionally, modulation of hemoglobin and NO release enhances oxygenation and microcirculation, contributing to improved metabolic balance, better glycemic control, and neuroprotection. Abbreviations: AKT—protein kinase B (PKB), CAT—catalase; CCL-1—C-C motif chemokine ligand 1; CCL-17—C-C motif chemokine ligand 17; GSH—glutathione; Hb—hemoglobin; HbO_2_—oxyhemoglobin; IκBα—inhibitor of nuclear factor kappa B alpha; IKK—IκB kinase; IL-1β—interleukin-1 beta; IL-6—interleukin-6; IL-10—interleukin-10; IRS-1—insulin receptor substrate-1; LLLT—low-level laser therapy; M1—classically activated (pro-inflammatory) macrophage phenotype; M2—alternatively activated (anti-inflammatory/repair) macrophage phenotype; NO—nitric oxide; PI3K—phosphoinositide 3-kinase; PIP_2_—phosphatidylinositol 4,5-bisphosphate; PIP_3_—phosphatidylinositol 3,4,5-trisphosphate; RNS—reactive nitrogen species; ROS—reactive oxygen species; SOD—superoxide dismutase; TLR4—toll-like receptor 4; VEGF—vascular endothelial growth factor. ↑—increase in expression/activity/level; ↓—decrease in expression/activity/level. This figure was created using Servier Medical Art (available at https://smart.servier.com/) (accessed on 1 May 2025).

The anti-inflammatory, antioxidant and metabolic regulatory mechanisms of photobiomodulation in diabetes are presented in Figure 6.

Furthermore, neuroprotective effects have been observed in models of brain inflammation, spinal cord injury, and neurodegeneration [202,203]. These anti-inflammatory and neuroprotective actions provide a mechanistic basis for the use of PBMT in treating both metabolic and neurological complications of diabetes [204]. Emerging evidence also suggests that PBMT may enhance neurogenesis and synaptic plasticity, offering potential benefits for cognitive impairment in diabetes [205,206].

### 8.4. Mast Cells Are Another Important Cellular Target

As they are reservoirs of histamine, proteases, and cytokines, mast cells play a pivotal role in orchestrating inflammation, angiogenesis, and wound repair. PBMT modulates mast cell degranulation through mitochondrial signalling pathways, thereby influencing histamine release and angiogenic factor expression [207,208]. This regulation improves local vascular responses and maintains tissue homeostasis while limiting excessive inflammation. In diabetic wounds, where mast cell dysregulation contributes to delayed healing, PBMT may restore balanced degranulation and promote effective tissue regeneration [208,209].

Influence on cellular proliferation and tissue regeneration: LLLT promotes cellular proliferation and tissue regeneration, both of which are essential for effective wound healing in diabetes patients [69]. PBMT increases the availability of ATP and modulates NO-dependent signalling, thereby stimulating fibroblast proliferation, keratinocyte migration, and angiogenesis [125]. Clinical and experimental studies have documented enhanced growth factor expression, including VEGF and fibroblast growth factor (FGF), as well as stem cell activation, following PBMT [113,210]. These effects are mediated through redox-sensitive transcription factors and mitochondrial signalling cascades, which coordinate cellular responses to injury and metabolic stress [157].

These reparative processes are particularly relevant in the management of diabetic foot ulcers, as PBMT accelerates wound closure, reduces the risk of infection, and improves functional tissue recovery [211,212]. As well as promoting re-epithelialisation and collagen synthesis, PBMT has been shown to enhance neovascularisation and restore extracellular matrix integrity, both of which are often compromised in chronic diabetic wounds [112,185,213]. While LLLT exerts local effects, systemic improvements in inflammatory balance and vascular function have also been observed, highlighting its dual role in localised tissue repair and systemic metabolic regulation [131].

However, the risk of tissue damage when parameters are not selected appropriately emphasises the necessity of standardising treatment protocols [214]. Key variables, such as wavelength, pulse duration, and treatment repetition rate or frequency, must be carefully calibrated to avoid overstimulation or thermal injury, particularly in vulnerable diabetic tissue [80]. Further research is needed to refine therapeutic parameters, confirm long-term results, and ensure safety in clinical practice. Randomised controlled trials and multicentre studies are essential for establishing evidence-based guidelines and integrating PBMT into mainstream diabetic wound care.

### 8.5. Modulatory Effects of Low-Level Laser and LED Therapy on Blood and Vascular Function in Diabetes

Recent studies have highlighted the effects of PBMT on haematological parameters. Reported changes include alterations in erythrocyte morphology, haemoglobin levels, and haematocrit [215,216]. These modalities also modulate immune cell function, cytokine production, and oxidative stress responses, thereby influencing inflammatory pathways and immune surveillance [74,217]. These effects are particularly relevant in the context of diabetes, where chronic inflammation and oxidative imbalance can lead to vascular dysfunction and impaired tissue perfusion [198]. Current research focuses on elucidating the underlying mechanisms, optimising treatment protocols, and exploring novel applications in haematology and transfusion medicine [133].

A key marker underlying the systemic effects of laser therapy is haemoglobin (Hb). Laser irradiation has been shown to induce conformational changes in haemoglobin, promoting the transition from the deoxy- to oxy-form (HbO_2_). During this process, the oxygen-binding affinity of haemoglobin is modulated by NO levels, with haemoglobin acting as a potential reservoir for NO. When exposed to lasers, Hb-nitrosyl complexes can undergo photolysis, releasing free NO, which is a potent vasodilator [218,219]. This facilitates the release of oxygen from Hb, enhances tissue oxygenation, and supports metabolic and enzymatic activity at the cellular level. These changes promote tissue repair and regeneration and improve overall physiological function. This illustrates a direct mechanistic link between laser therapy and enhanced oxygen delivery in biological tissues [220]. This mechanism may help to counteract hypoxia in diabetic tissues, a major contributor to delayed wound healing and neuropathy [69,221].

Furthermore, it has been demonstrated that PBMT induces pro-apoptotic responses in a dose-dependent manner. When administered at the correct frequencies, higher-energy doses elevate intracellular calcium levels and generate ROS within mesenchymal stem cells [217]. These findings emphasise the importance of carefully calibrating PBMT and LED parameters to maximise therapeutic effects and minimise potential oxidative stress. Inappropriate dosing can reduce efficacy and exacerbate cellular damage, particularly in tissues compromised by diabetic pathology [80].

In our recent studies, we investigated the effects of low-intensity infrared laser irradiation and red LED light on oxidative stress markers in erythrocytes [222,223]. Our results suggest that both modalities significantly reduce lipid peroxidation levels, indicating their potential to mitigate oxidative damage in blood cells. Furthermore, we assessed the time- and dose-dependent effects of low-intensity infrared irradiation on lipid peroxidation biomarkers. This provided additional confirmation of the modulatory effects of these therapies on oxidative stress parameters [222,223]. Together, these results support the hypothesis that PBMT and LED therapy can restore redox balance in diabetic blood cells, thereby enhancing their stability, oxygen-carrying capacity, and survival.

In summary, LLLT and LED therapy are safe and effective treatments for improving vascular function, oxygen delivery, and haematological parameters in patients with DM. Their multifaceted cellular effects, ranging from haemoglobin conformation modulation to immune response regulation, support their potential integration into comprehensive diabetes management strategies. Due to their non-invasive nature and minimal side effects, these therapies could be valuable additions to pharmacological treatment, especially for patients with poor vascular access or contraindications to conventional therapies. Further research is needed to refine protocols and expand their use in clinical practice.

## 9. Laser Therapy for Glycaemic Control

The use of laser therapy to improve glycaemic control has gained increasing attention as a potential complementary approach to the treatment of DM. PBMT has been reported to have beneficial effects on glucose metabolism, including reducing inflammation, modulating oxidative stress, and improving mitochondrial function [174]. These cellular processes are directly linked to enhanced insulin sensitivity and improved glycaemic stability, both of which are critical targets in diabetes management [73]. Notably, these effects may mitigate insulin resistance at the cellular level, particularly in skeletal muscle and adipose tissue, which are pivotal sites of glucose uptake [174].

Furthermore, laser therapy can indirectly influence glucose metabolism by promoting vascularisation, stimulating mitochondrial biogenesis, and activating intracellular signalling cascades involved in insulin regulation, including the PI3K/Akt pathway [72,224]. PBMT can counteract some of the adverse effects of chronic hyperglycaemia, such as impaired wound healing and vascular dysfunction, by facilitating tissue repair and regeneration [225,226]. These mechanisms also contribute to improved endothelial function, which plays a central role in maintaining glucose homeostasis and preventing diabetic complications [227]. Together, these findings emphasise the multifaceted potential of PBMT as a complementary therapy for optimising glycaemic control.

Experimental studies in animal models have provided encouraging insights into the role of PBMT in glycaemic regulation. However, the specific PBMT parameters that are critical for reproducibility and efficacy, such as wavelength, dose, and pulse duration, vary widely among studies. For instance, in diabetic rodents, laser irradiation applied to pancreatic tissue or skeletal muscle has been demonstrated to lower fasting blood glucose levels, enhance glucose tolerance, and improve insulin sensitivity [228,229,230]. Furthermore, PBMT has been linked to the increased expression of the glucose transporter GLUT4 in muscle tissue, indicating a direct impact on glucose uptake pathways [174,226]. This upregulation of GLUT-4 is particularly significant, as it represents a key insulin-dependent mechanism for clearing glucose from the bloodstream. These findings suggest that PBMT may have a multifaceted effect on glycaemic control. However, standardisation of treatment protocols is required before these results can be applied to clinical settings.

Although limited, evidence from small-scale human studies has also suggested potential benefits. Clinical investigations have reported modest reductions in fasting plasma glucose and HbA1c levels in patients with type 2 diabetes following PBMT protocols [226,231]. Improvements in peripheral insulin sensitivity and endothelial function have also been observed, suggesting that PBMT could complement standard pharmacological therapy by simultaneously targeting metabolic and vascular mechanisms [227]. However, as these clinical trials vary in design with respect to wavelength, energy density, irradiation site, and treatment duration, cross-study comparisons are difficult [231]. Furthermore, differences in patient characteristics, such as age, disease duration, and comorbidities, may influence treatment outcomes and should be considered in future studies.

Although the preliminary data are promising, the current evidence base is insufficient to establish PBMT as the standard therapy for glycaemic control. Several limitations must be acknowledged. Firstly, most studies are preclinical or involve small patient cohorts, which limits generalisability [226]. Secondly, heterogeneity in laser parameters and treatment protocols contributes to conflicting outcomes across studies. For instance, while some trials report significant reductions in fasting glucose or HbA1c levels, others find no statistically significant changes [231]. This inconsistency highlights the need for harmonised methodologies and consensus on therapeutic dosing guidelines.

In addition, the long-term safety and sustainability of the glycaemic effects of PBMT remain poorly understood. Most available studies assess outcomes over periods ranging from weeks to months, and there is limited data on the effects of long-term use in diabetic populations [232,233]. Another unresolved issue is the determination of the optimal irradiation parameters, including wavelength, energy density, frequency, and target tissue, to maximise therapeutic efficacy while minimising risks [73]. Finally, the variability in clinical results may be due to placebo effects and difficulties in blinding patients to PBMT interventions [73]. To address these challenges, future trials should incorporate rigorous blinding procedures, placebo controls, and stratified patient selection to ensure robust and reproducible outcomes.

Overall, however, PBMT represents a novel non-invasive strategy with the potential to enhance insulin sensitivity and improve glycaemic regulation in preclinical models and small-scale human studies [226]. By modulating mitochondrial function, oxidative stress, inflammation, and vascular responses, laser therapy may offer multifactorial benefits in diabetes management [233]. Nevertheless, the heterogeneity of the available studies as well as their limited sample sizes and conflicting results emphasise the necessity for large-scale standardised clinical trials to confirm efficacy, establish optimal treatment protocols, and ascertain long-term safety [73,232]. Until such evidence is available, PBMT should be considered a promising experimental adjunctive intervention in glycaemic control management to be used within research settings or alongside established therapies.

## 10. Laser Therapy for Managing Diabetes Complications

### 10.1. Laser Therapy in Diabetic Neuropathy

Diabetic peripheral neuropathy (DPN) is one of the most common and debilitating complications of diabetes mellitus. It is characterised by progressive nerve damage, sensory disturbances, and chronic neuropathic pain. These symptoms can result in substantial disability and a diminished quality of life [234]. Current pharmacological approaches primarily address the symptoms, providing only partial relief and highlighting the need for innovative therapeutic strategies. PBMT is a non-invasive treatment that has shown promise in alleviating neuropathic symptoms and improving nerve function [226,235]. Its favourable safety profile and ease of application make it a particularly attractive option for long-term management in outpatient settings.

Clinical and experimental evidence suggests that LLLT can contribute to pain relief and improved electrophysiological outcomes in DPN. By enhancing microcirculation and reducing oxidative stress in peripheral nerves, LLLT improves nerve conduction velocity and reduces abnormal spontaneous activity—the underlying cause of neuropathic pain [236]. Notably, clinical studies have repeatedly demonstrated reductions in pain perception and enhancements in nerve conduction velocity in patients undergoing laser therapy, as opposed to those receiving placebo treatment [237]. These improvements are often accompanied by enhanced tactile sensitivity and reduced paresthesia, which are key indicators of functional nerve recovery.

Randomised controlled trials and small-scale clinical studies provide supportive evidence for the effectiveness of LLLT in improving sensory nerve function and patient-reported outcomes [153,238]. Notably, dynamic thermolaser therapy (DTLT), a variant of LILT, has been associated with significant improvements in mobility, sleep quality, and daily living activities among patients with DPN [239]. Furthermore, pain reduction in the DTLT group was significantly greater and more sustained than in the sham treatment group, suggesting long-lasting therapeutic benefits. Such sustained effects are particularly valuable in such chronic conditions as DPN, where symptom recurrence is common and long-term relief is difficult to achieve. These studies suggest that laser therapy alleviates neuropathic pain and contributes to functional improvements, thereby enhancing patients’ quality of life.

The therapeutic effects of LLLT in DPN are mediated through several complementary mechanisms. Firstly, LLLT promotes nerve cell survival and axonal regeneration by stimulating mitochondrial function and supporting bioenergetic recovery in damaged nerves [226]. Secondly, anti-inflammatory effects are demonstrated by a reduction in pro-inflammatory mediators, such as monocyte chemoattractant protein-1 (MCP-1) and interleukin-6 (IL-6), which helps to reduce the inflammatory response that drives neuropathic progression [153]. Thirdly, LILT stimulates angiogenesis and enhances the local blood supply, further supporting neuroprotection and regeneration [239]. Furthermore, modulation of neurotransmitter release and activity within pain signalling pathways contributes to reduced pain perception and improved sensory function [238]. Furthermore, emerging evidence suggests that LLLT may influence Schwann cell activity, which is critical for peripheral nerve repair and remyelination [236].

Overall, the current body of evidence suggests that low-intensity laser therapy is a safe and non-invasive way of managing diabetic neuropathy that is potentially effective. By improving nerve conduction, reducing pain perception, stimulating neuroregeneration, and alleviating inflammation, laser therapy provides benefits that go beyond merely alleviating symptoms. However, optimising treatment parameters, conducting long-term efficacy studies, and clarifying variability in patient responses are essential for its broader clinical adoption. Standardisation of protocols, including wavelength, fluence, pulse duration, and treatment frequency, is necessary to ensure reproducibility and clinical reliability [80]. As mechanistic research continues to advance, LILT shows promise as an innovative adjunctive modality that could mitigate neuropathic complications and contribute to improved metabolic outcomes in patients with diabetes [153,226].

### 10.2. Laser Therapy for Diabetic Foot and Wound Healing

Chronic foot ulcers are among the most severe and costly complications of diabetes mellitus. They often lead to infection, amputation, and an increased risk of death [240]. Impaired wound healing in diabetic patients is caused by persistent hyperglycaemia, vascular dysfunction, neuropathy, and altered immune responses, all of which hinder tissue repair [241]. LLLT has emerged as a promising complementary approach to improving outcomes in the management of diabetic foot ulcers by targeting key molecular and cellular pathways [226,242]. Its non-invasive nature and minimal side effects also make it suitable for patients with comorbidities or contraindications to pharmacological interventions.

Both experimental and clinical studies have demonstrated that LLLT can accelerate the healing of diabetic ulcers via various mechanisms. Laser therapy enhances angiogenesis by upregulating VEGF expression and promoting endothelial cell proliferation, thereby improving the blood supply to ischaemic tissues [243]. Increased angiogenesis contributes to greater oxygen and nutrient delivery, which is crucial for tissue repair. This effect is particularly important in diabetic wounds, where microvascular impairment often limits perfusion and delays healing [112].

In parallel, LLLT stimulates fibroblast proliferation and collagen synthesis, thereby supporting extracellular matrix remodelling and wound closure [242]. Photobiomodulation has also been shown to modulate inflammatory responses by reducing pro-inflammatory cytokine expression while enhancing anti-inflammatory signalling. Together, these effects create a microenvironment that promotes regeneration, resulting in faster re-epithelialisation and stronger tissue integrity [244]. Furthermore, LLLT may influence keratinocyte migration and epithelial barrier restoration, both of which are essential for the recovery of functional skin [245].

Clinical studies in patients with diabetic foot ulcers suggest that LLLT improves healing rates when combined with standard wound care [226,246]. Randomised controlled trials and case series have reported shorter healing times, reduced ulcer size, and a decreased risk of infection in patients treated with laser therapy, compared to those treated with conventional therapy alone [247]. Furthermore, laser therapy has been associated with reduced pain at ulcer sites, improved local circulation, and enhanced granulation tissue formation. Importantly, these benefits have also been observed in chronic, non-healing ulcers, which are usually difficult to treat [245]. Such outcomes are clinically significant as chronic ulcers are a major predictor of lower limb amputation and prolonged hospitalisation.

The modalities tested included DTLT and low-intensity diode lasers, with the treatment protocols varying in terms of wavelength, energy density, and duration. Despite the heterogeneity of the protocols, consistent improvements in outcomes support the clinical relevance of LLLT as an adjunctive intervention in the treatment of diabetic foot conditions [245]. However, standardisation of these protocols is essential to ensure reproducibility and facilitate broader clinical adoption [80].

Nevertheless, laser therapy should not be considered a replacement for standard wound care in the management of diabetic wounds but rather a complementary treatment. Standard care strategies, such as debridement, infection control, offloading, and optimal glycaemic regulation, remain essential. Integrating LLLT into multidisciplinary treatment protocols could enhance the overall effectiveness of care by addressing the cellular dysfunction and impaired angiogenesis that underlie poor wound healing in diabetes [226]. The combination of laser therapy and established interventions has the potential to synergistically improve healing rates, reduce the risk of amputation, and enhance patients’ quality of life. To achieve this, collaboration between diabetologists, wound care specialists, and rehabilitation teams is crucial. Further research is needed to optimise treatment parameters, evaluate long-term outcomes, and develop evidence-based guidelines for its widespread clinical implementation [246].

### 10.3. Laser Therapy for Diabetic Retinopathy

Diabetic retinopathy (DR) is one of the leading causes of vision impairment and blindness among working-age adults worldwide. It is characterised by progressive microvascular changes in the retina, including capillary occlusion, microaneurysms, haemorrhages, and pathological neovascularisation, which are driven by hypoxia and increased VEGF expression [248]. Over the past few decades, ophthalmic laser therapy has played a pivotal role in managing DR; however, newer therapeutic approaches have significantly altered its application [13]. Notably, advancements in laser technology have shifted the focus from destructive coagulation to the selective modulation of retinal physiology, enabling more precise and patient-specific interventions.

Conventional panretinal photocoagulation (PRP), introduced in the 1970s, was the first major breakthrough in reducing the risk of severe vision loss in proliferative diabetic retinopathy (PDR). PRP involves applying thermal laser burns to the peripheral retina to reduce hypoxia-induced VEGF release and subsequent neovascularisation. Large multicentre clinical trials, such as the Diabetic Retinopathy Study and the Early Treatment Diabetic Retinopathy Study, established PRP as the gold standard for decades by demonstrating significant reductions in severe visual loss [13].

Despite its efficacy, PRP is associated with significant adverse effects, such as peripheral visual field loss, impaired night vision, and exacerbation of macular oedema. Consequently, it is now primarily used for advanced cases of PDR, while new therapeutic strategies aim to minimise collateral retinal damage [73,226]. This shift reflects a broader trend in ophthalmology towards preserving retinal architecture and function, particularly in patients with early-stage disease or concurrent macular involvement.

Technological advances have led to the development of subthreshold micropulse laser therapy (MPLT) and other modified approaches designed to minimise retinal damage while maintaining therapeutic efficacy [249,250]. Unlike conventional photocoagulation therapy, which relies on visible thermal burns, the micropulse laser delivers energy in short, repetitive pulses separated by cooling intervals. This prevents thermal coagulation and limits structural damage. It also enables selective stimulation of the retinal pigment epithelium and modulation of inflammatory and angiogenic pathways while avoiding scarring [250]. MPLT has also been shown to preserve contrast and retinal sensitivity, both of which are critical for maintaining functional vision in daily activities [251].

Clinical studies suggest that microperimetry-guided laser treatment (MPLT) is an effective treatment for diabetic macular oedema (DME). It improves visual acuity, reduces retinal thickness, and preserves retinal sensitivity [251]. Other innovations, such as navigated laser systems and pattern-scanning laser photocoagulation, enhance precision further and reduce treatment-associated morbidity [226]. These systems enable real-time eye tracking and automated delivery of laser spots, thereby improving consistency and reducing operator-dependent variability.

The introduction of intravitreal anti-VEGF agents, such as ranibizumab, aflibercept, and bevacizumab, has transformed the management of diabetic retinopathy (DR) and DME by shifting the therapeutic paradigm away from an exclusive reliance on laser therapy. Anti-VEGF therapy directly targets the molecular driver of pathological angiogenesis and vascular leakage, leading to superior visual acuity outcomes in patients with DME, compared to laser therapy [249]. However, anti-VEGF treatment requires repeated intravitreal injections, long-term patient adherence, and careful monitoring, all of which can be burdensome and costly. In settings with limited resources or among patients with poor adherence, laser therapy may be a more practical and sustainable alternative.

In contrast, laser therapy usually requires fewer interventions and can achieve long-lasting stabilisation, particularly when combined with pharmacological therapy [250]. Current clinical practice increasingly favours an integrative approach in which anti-VEGF therapy is the primary treatment for DME, and modified laser strategies are used to reduce injection frequency and improve long-term outcomes [251]. Contemporary practice positions laser therapy as a complementary strategy to pharmacological treatment, contributing to a personalised multimodal approach to preserving the vision of patients with diabetes [226]. Future research should focus on identifying biomarkers that predict response to laser therapy, enabling more precise patient selection and optimisation of outcomes.

### 10.4. Dental and Periodontal Applications in Diabetes

Diabetes mellitus is a systemic metabolic disorder that significantly increases the risk of periodontal disease and exacerbates its severity. Hyperglycaemia leads to impaired neutrophil function, altered collagen metabolism, and elevated levels of pro-inflammatory cytokines, all of which contribute to the destruction of periodontal tissue [252,253]. Therefore, effective periodontal management is crucial for both oral health and systemic glycaemic control in diabetic patients [254]. Poor periodontal health has also been associated with increased insulin resistance, highlighting the bidirectional relationship between oral and systemic inflammation [255].

Diode lasers (wavelength: 800–980 nm) have become widely accepted in periodontal therapy due to their affinity for pigmented tissues and bactericidal properties. They are particularly effective in reducing periodontal pocket depths and controlling inflammation in diabetic patients [256,257]. Studies indicate that combining diode laser therapy with conventional scaling and root planing (SRP) significantly reduces microbial loads and inflammatory markers in gingival tissue [258,259]. Furthermore, meta-analyses demonstrate that, in patients with type 2 diabetes and periodontitis, SRP combined with diode laser therapy results in more substantial reductions in probing depth and HbA1c levels than SRP alone [260,261]. The photobiomodulation properties of diode lasers also promote tissue healing and reduce postoperative discomfort, which benefits patients with impaired wound healing associated with diabetes [262]. This is particularly important for diabetic patients, who commonly experience delayed healing and an increased risk of infection.

Er:YAG lasers (wavelength: 2940 nm) are highly effective in ablating both hard and soft tissue while causing minimal thermal damage. When used to treat periodontitis, they allow subgingival calculus and diseased epithelium to be removed precisely while preserving healthy periodontal structures [263,264]. Clinical trials in diabetic patients have shown that Er:YAG-assisted periodontal therapy improves clinical attachment levels and reduces probing depths more effectively than SRP alone [173]. Furthermore, Er:YAG lasers stimulate fibroblast proliferation and collagen synthesis, thereby supporting periodontal regeneration in patients with metabolic compromise [265,266]. Their ability to modulate inflammatory cytokines and enhance tissue oxygenation makes them particularly suitable for managing chronic periodontal inflammation in diabetic patients.

CO_2_ lasers (wavelength: 10,600 nm) provide an effective means of ablating soft tissue and achieving haemostasis. Their high absorption by water-rich tissues enables precise debridement of the gingiva and periodontal pockets [267]. Several clinical reports and trials [66,268] have shown that, when applied as laser curettage or as an adjunct to subgingival CO_2_ debridement alongside SRP in diabetic patients, CO_2_ laser therapy reduces gingival inflammation and bleeding on probing and enhances pocket depth reduction, compared to SRP alone. CO_2_ treatment also achieves good haemostasis and may reduce recolonisation of the pockets by microbes after surgery, which is important for long-term stability in hyperglycaemic conditions [269]. Although there is less literature on CO_2_ lasers than on diode or Er:YAG lasers, randomised controlled clinical studies support their beneficial role as an adjunct in periodontal therapy, also for patients with systemic conditions that impair healing, such as diabetes [66,268]. Their haemostatic properties are particularly advantageous in patients with vascular fragility or on anticoagulant therapy, both of which are common in diabetic populations.

Furthermore, regular periodontal maintenance involving laser therapy in diabetic patients has been shown to reduce the risk of disease progression, improve oral hygiene outcomes, and enhance patient comfort and compliance [254,255]. The synergy between laser therapy and glycaemic control emphasises the importance of integrated management strategies that consider periodontal health to be an integral part of systemic disease management [255,270]. Collaboration between diabetologists and dental professionals is vital to ensure coordinated care and optimal patient outcomes.

Thus, laser technologies—including diode, Er:YAG, and CO_2_ lasers—offer considerable advantages in the periodontal treatment of diabetic patients. Their ability to reduce the microbial load, modulate inflammation, and promote tissue regeneration can contribute to improved periodontal health and potentially positively impact glycaemic control. Incorporating laser-assisted therapies into routine periodontal management protocols for diabetic patients is a promising strategy for improving both oral and systemic health. Future research should focus on long-term outcomes, cost-effectiveness, and patient-reported outcomes to support broader clinical implementation.

## 11. Safety Profile and Adverse Effects

Thanks to its non-invasive nature and ability to promote tissue healing, LLLT is widely used in medicine, dentistry, and ophthalmology. When used according to established protocols, it is generally considered safe, with a low incidence of serious adverse effects. This is primarily because low energy densities are used to stimulate cellular activity without inducing thermal damage or tissue necrosis [73,236,271]. The therapeutic window of LLLT—defined by specific ranges of wavelength, fluence, and exposure time—is designed to maximise biological effects while minimising risk, making it suitable for repeated use in chronic conditions.

Numerous clinical studies have confirmed that LLLT using diode, Er:YAG, and CO_2_ lasers at therapeutic doses is generally well tolerated. Common mild adverse effects are transient and may include localised erythema, mild pain, or temporary oedema at the site of irradiation. Systemic toxicity has not been reported, and repeated sessions are generally considered safe for long-term therapy. However, patient monitoring and adherence to the manufacturer’s recommended energy parameters are essential to maintain this favourable safety profile [73,236,272,273]. It is also recommended that treatment parameters and patient responses are documented to ensure traceability and support clinical decision-making.

Although lasers are generally safe, certain applications carry specific risks. In ophthalmology, for example, using lasers improperly near the retina can result in photochemical or thermal injury, which can lead to impaired vision. To prevent such complications, protective measures must be taken, such as wearing appropriate eye shields and strictly controlling exposure duration and intensity [273,274]. Laser devices used in ophthalmology must also comply with international safety standards to ensure ocular protection.

In dentistry, potential risks include thermal damage to the dental pulp or surrounding soft tissues if high-powered lasers are misused. Additionally, inadvertent exposure to the oral mucosa or eyes can cause localised burns or transient discomfort. Proper training, adherence to recommended power settings, and use of protective eyewear for both patients and clinicians are essential to minimise these risks [271,275]. Operator experience and device calibration are critical in preventing iatrogenic injury, particularly in anatomically sensitive regions.

Contraindications for LLLT include malignancies in the treatment area, active haemorrhaging, and known photosensitivity disorders. Caution should also be exercised when treating pregnant patients, particularly if laser exposure could affect the abdomen or pelvic region. Patients with epilepsy require special consideration, as certain laser wavelengths could potentially trigger photic seizures [276,277]. In such cases, alternative therapies should be considered and an interdisciplinary consultation may be necessary.

Precautions should also be taken to carry out a thorough patient assessment to identify any conditions that could increase the risk of adverse effects. To maintain therapeutic efficacy while avoiding tissue damage, clinicians must ensure that the devices are accurately calibrated, the correct wavelength is selected, and the exposure times are appropriate. Standardised pre-treatment screening protocols and informed consent procedures should be implemented to enhance patient safety and legal compliance.

It is essential to clearly distinguish between classical retinal photocoagulation (e.g., panretinal photocoagulation, PRP) and photobiomodulation therapy (PBMT/LLLT), as these approaches differ fundamentally in their mechanisms of action, energy parameters, and biological effects. Panretinal photocoagulation (PRP), commonly used in proliferative diabetic retinopathy, relies on the thermal and destructive effects of high-energy laser radiation. The procedure intentionally induces controlled retinal burns in order to reduce ischemia-driven neovascularisation. Its mechanism is based on tissue coagulation, photothermal damage, and partial retinal ablation [226]. In contrast, PBMT/LLLT operates within a non-thermal, low-energy range, typically below 500 mW, and does not induce tissue destruction. Its biological effects are mediated through photochemical and photobiological mechanisms, primarily involving mitochondrial chromophore activation, modulation of reactive oxygen species, nitric oxide release, and regulation of inflammatory pathways. Rather than causing coagulation or ablation, PBMT aims to restore cellular homeostasis and enhance physiological repair mechanisms [80,199,200]. Therefore, although both interventions use laser light, they represent fundamentally different therapeutic paradigms: one ablative and tissue-destructive (PRP), the other modulatory and bio-stimulatory (PBMT/LLLT) (Table 5). Clear terminological differentiation is necessary to prevent interpretative ambiguity, particularly in interdisciplinary contexts.

Low-level laser therapy has a strong safety profile for medical, dental, and ophthalmological applications. Adverse effects are generally mild and transient. It is essential to be aware of potential ocular and dental risks, adhere to contraindications, and carefully monitor patients to ensure that the treatment is both safe and effective. When applied correctly, LLLT is a minimally invasive therapeutic option with a low risk of complications [271,273,275]. Its favourable safety profile supports its integration into multidisciplinary care pathways, particularly for patients with chronic or systemic conditions requiring long-term management.

## 12. Future Applications of Laser Therapy

Due to its non-invasive nature, minimal side effects, and ability to stimulate biological processes at the cellular level, laser therapy has been widely adopted across various medical disciplines. These properties make it particularly attractive for the long-term management of chronic conditions where tissue preservation and regeneration are essential. As technology continues to evolve, the range of applications for laser therapy is expanding, especially in diabetes management, multimodal therapeutic strategies, and routine clinical practice.

In recent years, PBM, which includes LLLT, has attracted attention due to its potential to modulate inflammation, enhance microcirculation, and improve metabolic function in patients with diabetes [65,70]. The ability of laser therapy to influence mitochondrial activity, redox balance, and cytokine expression positions it as a promising adjunct in both preventive and interventional approaches to metabolic diseases. Furthermore, the development of portable and adaptable laser systems has made it more accessible in clinical settings and paved the way for personalised treatment protocols [250].

An additional important aspect is the growing body of clinical evidence confirming the safety and efficacy of laser therapy in diabetes care. Recent randomised controlled trials have demonstrated improvements in wound healing, reduction in neuropathic pain, and enhanced glycemic control, highlighting its potential as a complementary method to conventional pharmacological and lifestyle interventions [278,279]. This emphasises the importance of integrating laser-based therapies into multidisciplinary care models to optimise patient outcomes and ensure long-term benefits.

### 12.1. Emerging Technologies and Their Potential Significance in Diabetology

Recent advancements in the laser technology have led to the development of PBM techniques, which show considerable promise in managing diabetes and its complications. LLLT, a form of PBM, has been shown to improve glucose metabolism, enhance insulin sensitivity, and reduce oxidative stress, which are all key contributors to diabetic pathophysiology [65,70]. These effects are mediated through mitochondrial stimulation, modulation of redox-sensitive transcription factors, and improved cellular energy dynamics. Notably, LLLT has also been shown to influence the expression of glucose transporter proteins (e.g., GLUT4), which are crucial for insulin-mediated glucose uptake in skeletal muscle and adipose tissue [72]. Furthermore, emerging evidence suggests that LLLT may positively affect pancreatic β-cell survival and function, thereby supporting endogenous insulin secretion and improving glycaemic control [73,280].

In addition to their therapeutic applications, laser-based technologies are being investigated for use in non-invasive glucose monitoring. Among the most promising modalities under investigation are optical coherence tomography (OCT) and mid-infrared spectroscopy (MIRS). These approaches aim to enable continuous glucose monitoring without the need for invasive blood sampling, thereby improving patient compliance, reducing discomfort, and enhancing glycaemic control [73,281,282]. Such innovations could transform diabetes self-management, particularly for individuals requiring frequent monitoring, including those with type 1 or gestational diabetes.

Another area of growing interest is the role of PBM in healing diabetic wounds. Chronic wounds, particularly diabetic foot ulcers, are notoriously difficult to treat and can lead to infection, hospitalisation, and amputation [240]. Studies have demonstrated that laser therapy can accelerate wound closure by promoting angiogenesis, stimulating fibroblast proliferation, enhancing collagen synthesis, and modulating inflammatory responses [212,283]. PBM also improves microcirculation and oxygenation in ischaemic tissues, both of which are critical for effective wound healing in diabetic patients [69].

Thus, these emerging technologies highlight the expanding role of laser therapy in diabetology, not only as a treatment modality but also as a diagnostic and preventive tool. Continued research and clinical validation are essential for fully integrating these innovations into personalised diabetes care.

### 12.2. Laser Therapy in Conjunction with Pharmacotherapy and Physiotherapy

Integrating laser therapy with pharmacological and physiotherapeutic treatments offers new possibilities for improving the efficacy of diabetes care. Combination therapies ensure synergistic effects by targeting multiple physiological pathways simultaneously, offering a more comprehensive approach to managing metabolic dysfunction and its complications. For example, combining LLLT with anti-inflammatory or antioxidant drugs can enhance therapeutic outcomes, particularly in conditions characterised by chronic inflammation, such as diabetic neuropathy. Laser irradiation has been shown to enhance drug penetration and bioavailability, potentially reducing required dosages and minimising side effects [13,131]. This approach may also improve the pharmacodynamics of medications that target oxidative stress and endothelial dysfunction, which are prevalent in diabetes-related vascular complications. Recent studies suggest that LLLT may upregulate cellular transport mechanisms and transiently increase membrane permeability, facilitating more efficient drug delivery to target tissues [4,284,285]. Importantly, preliminary clinical evidence suggests that combining LLLT with standard antidiabetic medications can improve glycaemic control and reduce the progression of microvascular complications, highlighting its potential [280,286].

In musculoskeletal and neurological rehabilitation, laser therapy has been successfully incorporated into physiotherapeutic interventions to improve mobility, alleviate pain, and enhance muscle function. For diabetic patients, combining laser therapy with structured exercise regimens can improve peripheral circulation, reduce neuropathic pain, and promote nerve regeneration, which are all commonly impaired in diabetes [68]. This multimodal approach may also support glycaemic control by improving insulin sensitivity through enhanced muscle metabolism and vascular perfusion [65].

Emerging research is exploring laser-induced transdermal drug delivery systems, which enable the controlled release of medications such as insulin through the skin. These systems use laser pulses to create microchannels in the skin, enabling enhanced absorption of therapeutic agents and eliminating the need for injections. This method could improve pharmacokinetics, reduce discomfort associated with injections, and increase patient compliance, particularly among individuals requiring frequent insulin administration [67,287]. Such technologies are particularly promising for paediatric and geriatric populations, where needle aversion and fragile skin pose challenges to conventional drug delivery.

Thus, combining laser therapy with pharmacological and physiotherapeutic approaches represents a promising frontier in diabetes management. By leveraging the complementary mechanisms of each approach, clinicians may achieve better outcomes in terms of glycaemic control, pain reduction, wound healing, and functional recovery. However, further clinical trials are needed to establish standardised protocols and evaluate long-term safety and efficacy.

### 12.3. Prospects for Implementation of Laser Therapy in Clinical Practice

Despite its promising therapeutic potential, the widespread adoption of laser therapy in clinical practice, particularly in diabetology, faces several challenges. These include the need for standardised treatment protocols, cost-effectiveness, accessibility, and regulatory oversight. Overcoming these barriers is essential to ensure safe, effective, and equitable integration of laser-based interventions into mainstream healthcare.

Large-scale randomised controlled trials are crucial for validating the long-term efficacy and safety of laser therapy for diabetes and related conditions. Although numerous preclinical and pilot studies have demonstrated positive outcomes, robust clinical evidence is still limited. Standardised treatment protocols, including optimal wavelengths, fluence, dosimetry, and session frequency, must be established to ensure reproducibility and consistent clinical results across diverse patient populations [202,250]. Furthermore, stratified trials accounting for diabetes type, disease duration, and comorbidities are required to customise laser therapy to individual patient profiles. It is equally important to include quality-of-life measures and patient-reported outcomes in clinical evaluations, as these reflect the real-world impact of therapy and guide its integration into holistic care [80].

Although laser therapy provides a non-invasive alternative to pharmacological and surgical interventions, its accessibility may be limited by the high cost of laser devices, maintenance, and clinician training, particularly in settings with limited resources. To facilitate broader clinical application, advances in portable, user-friendly, and affordable laser systems are necessary [71,288]. Economic evaluations comparing laser therapy with standard care are also needed to inform reimbursement decisions and health policy.

Clear regulatory frameworks are essential for the safe and standardised use of laser therapy in clinical settings. Regulatory bodies must define approved indications, contraindications, safety thresholds, and documentation requirements. Collaboration between researchers and clinicians is vital in order to develop comprehensive guidelines that will support clinical adoption [80,289,290]. Furthermore, interdisciplinary cooperation between endocrinologists, physiotherapists, dentists, and biomedical engineers will be crucial in order to exploit the full therapeutic potential of lasers and ensure the delivery of integrated care.

To ensure safe and effective implementation of laser therapy, healthcare professionals must receive specialised training in laser physics, tissue interactions, and device operation. Incorporating laser therapy modules into medical and allied health curricula could accelerate clinical readiness. Furthermore, integrating laser therapy into electronic health records and clinical decision support systems could help to standardise documentation and monitor treatment outcomes [291].

In conclusion, the future of laser therapy in clinical practice, particularly in diabetes management, appears promising. Integrating cutting-edge laser technologies with pharmacotherapy, physiotherapy, and personalised care models could enhance treatment efficacy and patient outcomes. However, further research, clinical validation, and cost-effective innovations are necessary to facilitate its widespread implementation and ensure equitable access across healthcare systems.

## 13. Conclusions

Laser therapy, which encompasses PBMT and LLLT, offers a promising additional approach to managing diabetes mellitus and its complications. Its non-invasive nature, minimal side effects, and ability to target key pathophysiological mechanisms, such as mitochondrial dysfunction, oxidative stress, impaired insulin signalling, and chronic inflammation, make it a valuable tool in modern diabetology. Consistent improvements in wound healing, neuropathic pain relief, glycaemic control, insulin sensitivity, and vascular function have been demonstrated in experimental and clinical studies.

However, the lack of standardized treatment protocols (including wavelength, dosage, and duration) and the limited number of large-scale randomized controlled trials currently restrict its widespread adoption. Additionally, patient-specific factors, such as diabetes type, duration, and comorbidities, can influence treatment outcomes, highlighting the need for personalized approaches.

Future research should focus on developing personalized laser protocols, integrating laser therapy with pharmacological and physiotherapeutic interventions, and establishing evidence-based clinical guidelines. Continued innovation, interdisciplinary collaboration, and robust clinical validation have the potential to make laser therapy an integral and standard component of comprehensive diabetes care, improving patient outcomes and quality of life.

## Figures and Tables

**Table 1:** Suggested therapeutic parameter ranges for PBMT/LLLT in diabetes-related conditions (based on most frequently reported values in the literature [95,96,97,98,99,100,101,102,103,104,105,106,107,108,109,110,111,112,113,114,115,116,117]).

Clinical Target	Wavelength (nm)	Power Output	Energy Density (Fluence, J/cm^2^)	Mode	Exposure Time	Notes
Superficial diabetic wounds	630–685 nm	10–200 mW	1–5 J/cm^2^	CW or pulsed	30–120 s per point	Red light; optimal for epidermal/dermal penetration
Deep or infected ulcers	780–904 nm	50–500 mW	4–10 J/cm^2^	Pulsed preferred	60–180 s per point	Greater tissue penetration; improved microcirculation
Diabetic neuropathy (peripheral nerves)	808–980 nm	100–500 mW	8–30 J/cm^2^	Pulsed	60–180 s per point	Deeper penetration; analgesic and anti-inflammatory effects
Musculoskeletal pain in diabetes	780–1064 nm	200–1000 mW	10–50 J/cm^2^	CW or superpulsed	1–5 min	For deeper structures; monitor thermal effect
Pancreatic region (experimental models)	630–850 nm	10–300 mW	2–10 J/cm^2^	CW	30–120 s	Mostly animal studies; requires further clinical validation

Abbreviation: CW—continuous wave.

**Table 2:** Comparative analysis of laser therapy modalities (according to [4,104,105,122,123,124,125,126,127]).

Laser Therapy Type	Power/Emission Mode	Mechanism of Action	Clinical Applications	Essential Notes
Low-level laser therapy (LLLT)	1–500 mW; Continuous or pulsed	Photobiomodulation via cytochrome c oxidase activation; ATP synthesis; anti-inflammatory effects	Musculoskeletal pain, wound healing, dermatology, oral mucositis prevention	Non-thermal; safe for superficial tissues and mucosal surfaces
High-intensity laser therapy (HILT)	>500 mW to several watts; Continuous	Biostimulation + thermal effect; deep tissue penetration; increased blood flow and tissue relaxation	Chronic pain, arthritis, joint stiffness, rehabilitation	Requires precise dosing; ideal for deep structures and neuropathic pain
Superpulsed laser therapy	High peak power; Short pulses	Rapid energy delivery without heat buildup; stimulates microcirculation and regeneration	Sports injuries, chronic pain, post-operative recovery	Combines deep penetration with patient comfort; minimizes thermal risk
Pulsed laser therapy	Pulsed emission; (ns-μs range)	Controlled energy bursts; angiogenesis, cytokine modulation, ATP stimulation	Orthopedics, dermatology, sports medicine	Allows fine-tuning of pulse parameters: frequency, peak power, duty cycle
Hot laser therapy	High power; Continuous emission	Thermal response: collagen remodeling, increased elasticity, muscle relaxation	Physiotherapy, muscle spasms, chronic inflammation	Used primarily for deep heating; risk of overheating if misused

**Table 3:** Shared and type-specific mechanisms of PBMT/LLLT in different types of diabetes (according to [13,16,17,18,73,74]).

Mechanism/Biological Target	Common to All Types of Diabetes	More Relevant in T1DM	More Relevant in T2DM
Improved microcirculation (NO release, eNOS activation)	✓	✓	✓
Reduction in oxidative stress (ROS modulation, mitochondrial stabilisation)	✓	✓	✓
Enhanced ATP production	✓	✓	✓
Acceleration of wound healing	✓	✓	✓
Modulation of inflammatory cytokines (TNF-α, IL-6, MCP-1)	✓	✓ (autoimmune context)	✓ (chronic low-grade inflammation)
Immunomodulation (NF-κB, macrophage polarization)	✓	High relevance—autoimmune β-cell destruction	Moderate relevance
Protection of pancreatic β-cells	Possible	High relevance	Moderate relevance
Improvement of insulin sensitivity (PI3K/Akt pathway, GLUT4 translocation)	Limited	-	High relevance
Reduction in insulin resistance	Limited	-	High relevance
Modulation of adipose tissue metabolism	Limited	-	High relevance
Neuroprotection in peripheral neuropathy	✓	✓	✓

The checkmark (✓) indicates confirmed relevance or documented effect of PBMT/LLLT for the specified diabetes type.

**Table 4:** Changes in metabolic and clinical parameters following laser therapy in patients with diabetes.

No	Model (Animal/Human)	Laser Therapy Type, Study Description	Proposed Mechanism of Action	References
1	Adults with diabetic macular edema (DME), CRT < 400 μm, BCVA > 24 ETDRS (*n* = 266)	Subthreshold micropulse laser (SML) vs. standard laser (SL); SML required more retreatments than SL	Comparable efficacy to SL; preservation of retinal sensitivity with fewer adverse effects (noted better safety profile vs. conventional laser)	[172]
2	120 diabetic patients with trigeminal neuralgia	LLLT (LASER SCANNER PAGANI, 2014) vs. EMT vs. control (drugs only)	LLLT reduced pain intensity, improved compound action potentials; enhanced neuromuscular function and lowered recurrence	[67]
3	Streptozotocin-induced diabetic rats (*n* = 40, 10 weeks DM)	Near-infrared laser (0–16 J/cm^2^, 2 weeks)	Mitochondrial activation, oxidative stress reduction, restoration of cavernosal tissue, improved erectile function	[167]
4	44 patients with T2DM and periodontitis	Non-surgical periodontal therapy ± Er:YAG laser	Adjunctive Er:YAG improved clinical attachment levels and periodontal outcomes (reduced HbA1c levels noted at 3 months)	[173]
5	40 patients with painful diabetic peripheral neuropathy (12 weeks)	Deep tissue laser (980/810 nm, 80:20, 0.8 W/cm^2^) vs. sham	Reduced pain, improved QoL, decreased IL-6, TNF-α, RANTES, MCP-1	[22]
6	Preclinical murine models of T1DM/T2DM with muscle injury	PBMT (LED/laser), PROSPERO-registered review	Reduced oxidative stress, improved muscle regeneration, pro-angiogenesis, reduced myosteatosis	[16]
7	In vitro and in vivo T2DM insulin resistance models	PBMT, LLLT	Improved insulin sensitivity, ↓ inflammation, ↓ oxidative stress, modulation of gut microbiota	[174]
8	Streptozotocin-induced T2DM rats	Manual vs. laser acupuncture at EX-B3 (785 nm, 0.3 J, 5 mW, 60 s)	Improved Langerhans islets morphology, ↑ β-cell density; both safe, laser more effective	[175]
9	T2DM rats (STZ 1%)	Compound laser acupuncture-moxibustion (10.6 μm + 650 nm, BL20, BL23, SP6, 5×/week, 5 weeks)	Improved glucose tolerance, ↓ insulin resistance	[176]
10	Adults with poorly controlled diabetes and center-involving DME (*n* = 266)	577 nm SML vs. argon/532 nm SL	Comparable efficacy, fewer side effects, but higher retreatment rates	[177]
11	Patients with chronic diabetic foot ulceration	LILI (1440 mW, 4 J/cm^2^, 6 weeks) vs. HBOT	HBOT more effective in ↑ TcPO_2_ and accelerating healing; LILI showed adjunctive benefit	[178]
12	137 women (40–65 yrs, skin phototypes II-IV)	PBM (660 & 590 nm, 3.8 J/cm^2^, 10 sessions)	30% wrinkle reduction; safe in diabetes/keloids (anti-inflammatory rejuvenation)	[179]
13	Patients with chronic wounds	Er:YAG laser vs. sharp debridement	Better patient preference (52.9% vs. 35.3%), ↓ bacterial load, ↓ wound size	[180]
14	Diabetic foot & non-healing ulcers	PBMT (660, 800, 970 nm; 30 kJ)	68–99% wound area reduction, ↑ granulation tissue	[181]
15	Patients with DME	Short-pulse CW (PASCAL, 532 nm) vs. micropulse (810 nm)	Infrared micropulse showed better functional outcomes, ↓ edema	[182]
16	Human model—19 patients with type 2 diabetes mellitus and painful peripheral neuropathy	Pre-post observational design; 19 T2DM subjects; LLLT (632.8 nm, 660 nm & 850 nm), dose 3.1–3.4 J/cm^2^; 10 sessions (plantar, dorsal foot, popliteal fossa, fibular neck); outcome measures: VAS, MNSI, VPT, and skin temperature	LLLT improved microcirculation and nerve regeneration; significant reduction in pain (VAS: 6.47 → 1.21), improved neuropathic scores (MNSI: 5.52 → 2.71), decreased vibration perception threshold (32.68 → 24.84), increased skin temperature (30.01 °C → 31.75 °C); biostimulatory effect linked to cytokine release (IL-1α, IL-2, IFN-γ, TNF-α) and enhanced ATP production	[152]
17	Diabetic rat model (STZ-induced; 3 groups: Control, DM, DM + PBMT; *n* = 15)	PBMT with GaAlAs red laser (660 nm); in vitro: 8 J/cm^2^ on D1 cells; in vivo: 4 J/cm^2^ daily for 12 weeks applied to 3 mm calvarial bone defects	PBMT enhanced osteogenic differentiation of bone marrow stem cells (↑ calcium deposition, ↑ mineralization); improved bone regeneration in vivo (↑ bone volume fraction, ↑ bone matrix formation); partially restored healing capacity in diabetic rats; mechanism linked to ATP production, collagen synthesis, osteoblast activation, and reduced inflammation; BMP-2 expression unchanged at 12 weeks	[183]
18	Streptozotocin-induced diabetic rat model (*n* = 15; 10 diabetic, 5 control)	LED PBM, 850 nm, 48 mW, 22 s, 1.0 J applied to liver region; groups: Sham (*n* = 5), untreated diabetic (*n* = 5), PBM-treated diabetic (*n* = 5); evaluation with NMR spectroscopy at 600 MHz	PBM modulated systemic and hepatic metabolism in diabetic rats: ↓ glucose and glycogen signals, ↑ acetate signal, normalization of lipid metabolism. Findings suggest PBM restores hepatic metabolic balance through mitochondrial and enzymatic regulation	[184]
19	Rat (STZ-induced diabetic wounds)	PBMT combined with collagen-based amniotic membrane scaffold (CSAM); daily application for 8 days post-wounding	Reduction in inflammation (↓ neutrophils, IL-1β, TNF-α, NF-κB); enhanced fibroblast proliferation, collagen deposition, angiogenesis; upregulation of VEGF and bFGF; acceleration of wound closure	[185]
20	Human (umbilical vein endothelial cells, HUVECs) under hyperglycemic conditions (in vitro)	Photobiomodulation (660 nm, CW, 10 mW/cm^2^, 200 s, 0.84 Einstein)	Enhanced endothelial cell proliferation, migration, and tubulogenesis; mediated via PDGF, VEGF, and TGF-β1 signalling; modulation of MMP-2 and MMP-9 activity	[186]
21	Human (dermal fibroblasts, keratinocytes, endothelial cells) under hyperglycemic conditions (in vitro)	Photobiomodulation with near-infrared polychromatic light (600–1200 nm, 2.4 J/cm^2^/min, 3 min, alternate days for 7 days)	Enhanced proliferation and migration of fibroblasts and keratinocytes; increased fibronectin and collagen synthesis; improved endothelial tube formation; restoration of cellular functions impaired by hyperglycemia	[187]
22	Mouse (C57BL/6, STZ-induced diabetes)	LED therapy: RED (660 nm) and NIR (830 nm), separately and combined, 10 days over 2 weeks	Improved neurovascular function: increased intra-cavernous pressure, endothelial cell density, angiogenesis, pericyte recruitment, neural regeneration; upregulation of NGF, NT-3, BDNF, VEGF, eNOS, phosphorylated PI3K; reduced apoptosis and increased cell proliferation	[188]
23	Human (Type 2 diabetic patients with chronic periodontitis)	LLLT adjunct to scaling and root planing (SRP), 6 sessions over 3 months	Anti-inflammatory: reduced TNF-α in gingival crevicular fluid; improved glycemic control (HbA1c); enhanced periodontal clinical parameters (PI, GI, BOP, PPD, CAL)	[189]
24	In vitro (3T3-L1 preadipocytes/adipocytes)	PBMT, low-power light on insulin-resistant cells	Improves glucose metabolism; reduces triglyceride accumulation; modulates gene expression related to adipogenesis; activates PI3K/AKT signaling pathway, affecting insulin signaling and GLUT4 function	[190]

Arrows indicate direction of change: ↑—increase/improvement, ↓—decrease/reduction.

**Table 5:** Key differences between retinal photocoagulation and PBMT/LLLT (according to [80,199,200,226]).

Parameter	Retinal Photocoagulation (PRP)	PBMT/LLLT
Energy level	High	Low
Thermal effect	Yes (intentional)	No (non-thermal)
Tissue effect	Coagulation/ablation	Cellular modulation
Biological goal	Destroy ischemic retina to reduce neovascularisation	Improve cellular metabolism and repair
Mechanism	Photothermal	Photochemical/mitochondrial

## Data Availability

No new data were created or analyzed in this study. Data sharing is not applicable to this article.
